# Role of Smad Proteins in Resistance to BMP-Induced Growth Inhibition in B-Cell Lymphoma

**DOI:** 10.1371/journal.pone.0046117

**Published:** 2012-10-01

**Authors:** Kanutte Huse, Maren Bakkebø, Sébastien Wälchli, Morten P. Oksvold, Vera I. Hilden, Lise Forfang, May L. Bredahl, Knut Liestøl, Ash A. Alizadeh, Erlend B. Smeland, June H. Myklebust

**Affiliations:** 1 Section of Immunology, Oslo University Hospital, Oslo, Norway; 2 Centre for Cancer Biomedicine, University of Oslo, Oslo, Norway; 3 Department of Informatics, University of Oslo, Oslo, Norway; 4 Department of Medicine, Stanford University, Stanford, California, United States of America; Cedars-Sinai Medical Center, United States of America

## Abstract

Bone morphogenetic protein (BMP) expression and signaling are altered in a variety of cancers, but the functional impact of these alterations is uncertain. In this study we investigated the impact of expression of multiple BMPs and their signaling pathway components in human B-cell lymphoma. *BMP* messages, in particular *BMP7*, were detected in normal and malignant B cells. Addition of exogenous BMPs inhibited DNA synthesis in most lymphoma cell lines examined, but some cell lines were resistant. Tumor specimens from three out of five lymphoma patients were also resistant to BMPs, as determined by no activation of the BMP effectors Smad1/5/8. We have previously shown that BMP-7 potently induced apoptosis in normal B cells, which was in contrast to no or little inhibitory effect of this BMP in the lymphoma cells tested. BMP-resistance mechanisms were investigated by comparing sensitive and resistant cell lines. While BMP receptors are downregulated in many cancers, we documented similar receptor levels in resistant and sensitive lymphoma cells. We found a positive correlation between activation of Smad1/5/8 and inhibition of DNA synthesis. Gene expression analysis of two independent data sets showed that the levels of inhibitory Smads varied across different B-cell lymphoma. Furthermore, stable overexpression of Smad7 in two different BMP-sensitive cell lines with low endogenous levels of *SMAD7*, rendered them completely resistant to BMPs. This work highlights the role of Smads in determining the sensitivity to BMPs and shows that upregulation of Smad7 in cancer cells is sufficient to escape the negative effects of BMPs.

## Introduction

Bone morphogenetic proteins (BMPs) are members of the TGF-β family of cytokines and control cellular processes like proliferation, apoptosis, migration and differentiation in many cell and tissue types [1]. More than 20 different BMPs have been identified in mammals, and these are further divided into at least four subgroups based on their sequence similarities and functions: BMP-2/4, BMP-5/6/7/8a/8b, BMP-12/13/14 and BMP-9/10 [2]. BMPs play important roles during embryonic development and they regulate tissue homeostasis in adults [1]. Several studies have shown that BMPs can influence the hematopoietic system as they regulate development of hematopoietic stem cells [3], inhibit B- and T-cell lymphopoiesis [4], [5], and affect mature immune cells [6]. Exogenously added BMP-2,-4, -6 and -7 inhibit cell growth and plasma-cell differentiation in human B cells [7], [8]. Furthermore, an *in vivo* study has shown that the BMP-signaling machinery affects B-cell functions in mice [9]. Similar to TGF-β, the functional outcome of BMP stimulation is highly dependent on cell type and microenvironment conditions.

BMPs transduce their effects via two types of serine/threonine receptors which they bind with different affinities [1]. Type II receptors are constitutively active, whereas type I receptors require ligand binding, ligand-receptor oligomerization and transphosphorylation via type II receptors to be activated. The active type I receptors phosphorylate the receptor-regulated Smads (R-Smads): Smad1, Smad5 or Smad8, which together with Smad4 form a complex and move to the nucleus where they bind DNA and regulate transcription of target genes. The pathway is negatively regulated on multiple levels, e.g. by intracellular inhibitory Smads: Smad6 and Smad7 [1].

The role of TGF-β in cancer has been extensively studied. Whereas TGF-β often is a tumor suppressor in early stages of tumor development, it can have enhancing effects in advanced tumors. Tumor cells can evade the anti-tumor effects of TGF-β by acquiring alterations in the TGF-β signaling pathway, such as mutations in receptors or Smad4 and upregulation of inhibitory Smads [10]. Similarly, alterations in components of the BMP signaling pathway have been found in several types of cancer, demonstrating their importance during tumorigenesis [11]. Whereas some studies showed that BMPs can promote tumorigenesis and metastasis, others demonstrated that BMPs can have negative effects on cancer [12], [13], illustrating the context-dependence of BMP effects. Resistance to BMPs has also been shown in some cancers and the mechanisms are similar to those found in the TGF-β pathway [11]. For instance, impaired expression of BMP receptors and Smad4 has been found in colorectal cancer [14].

In this study we have investigated the intracellular signaling proteins of BMPs and the functional outcome of exogenously added BMPs in B-cell lymphoma, to study whether possible escape mechanisms are similar or different to other cancers. We found that also B-cell lymphoma cells could escape BMP inhibitory effects. However, the mechanism was not loss of BMP receptors, which is shown to occur in other cancers. Instead, we found that expression levels of inhibitory Smads varied across lymphoma cells, and that overexpression of Smad7 could transform highly BMP-sensitive cell lines to become BMP resistant.

## Results

### Expression of *BMP* mRNA in Normal and Malignant Germinal-center B Cells

**Figure 1:**
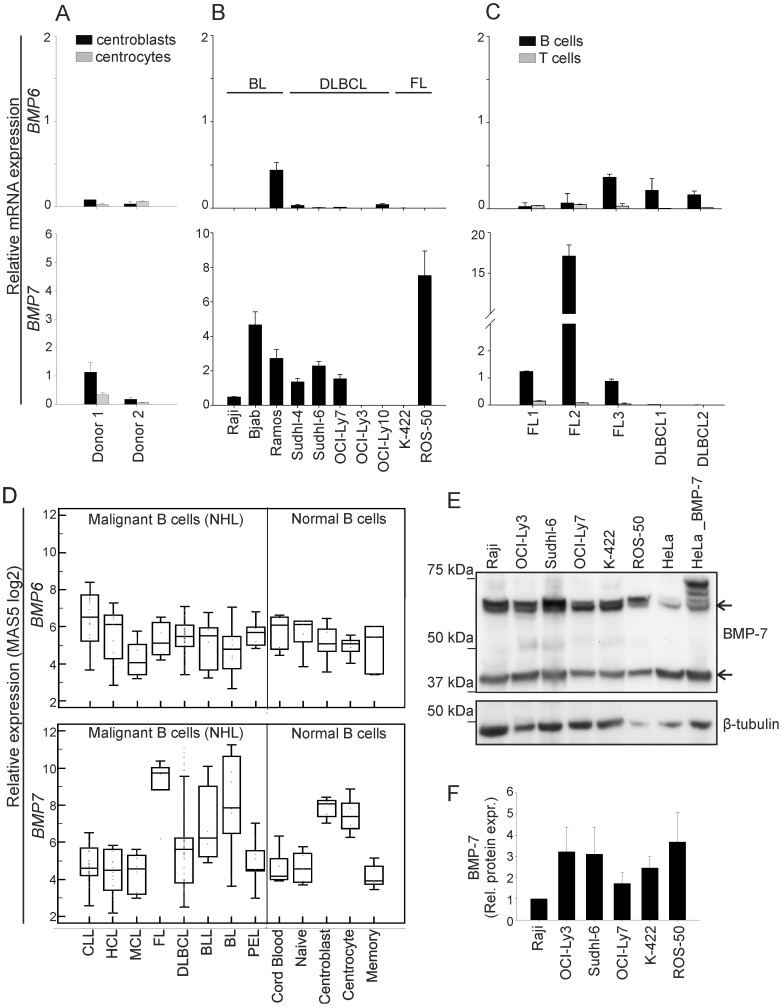
Normal and malignant B cells express *BMP6* and *BMP7*. *BMP* mRNA expression was determined by real-time RT-PCR, and the results are relative to the expression in human fetal brain tissue. (A) Centrocytes (CD19^+^CD38^+^IgD^−^CD77^-^) or centroblasts (CD19^+^CD38^+^IgD^−^CD77^+^) from normal human tonsils (relative expression ± SD of duplicates). (B) Lymphoma cell lines (means ± SEM, *n* = 3 for *BMP6*; *n* = 5 for *BMP7*). (C) Purified malignant B cells (CD20^+^CD10^+^Igκ/Igλ^+^) or tumor-infiltrating T cells (CD20^−^CD3^+^) from lymphoma patient samples (relative expression ± SD of duplicates). (D) Relative expression of *BMP6* and *BMP7* across non-Hodgkin’s lymphoma (NHL; left of line) and in normal B-cell populations (right of line) obtained from gene expression data set [16]. (E-F) Protein expression of BMP-7 was determined by Western blot analysis. Protein bands correspond to propeptide (lower band) and uncleaved full-length monomer (upper band). HeLa_BMP-7: Lysate from HeLa cells transfected with BMP-7. β-tubulin was used as loading control. One representative experiment (E) and mean values (F) of BMP-7 levels are shown (means ± SEM, *n* = 3).

Expression of BMP-6 has been detected in some lymphoma cell lines [15], but the expression of BMPs in adult lymphoid tissue is largely unknown. We therefore examined the expression of *BMP* mRNA in normal and malignant germinal-center B cells, by using real-time RT-PCR. FACS-sorted centrocytes and centroblasts from tonsils expressed *BMP7*, but only low levels of *BMP6* (Figure 1A). Studies in lymphoma cell lines of different subtypes showed that seven out of ten expressed *BMP7*, whereas three out of ten had detectable *BMP6* levels (Figure 1B). Only one cell line expressed *BMP4* (Figure S1), and *BMP2* mRNA was undetectable (data not shown).

Next, we used tumor samples from lymphoma patients and separated the malignant B cells from the infiltrating T cells by FACS sorting. *BMP6* was expressed at low to intermediate levels in all malignant B cells, whereas infiltrating T cells expressed undetectable to low levels of *BMP6* and *BMP7* (Figure 1C). Furthermore, malignant B cells from tumor samples of three out of three Follicular lymphoma (FL) patients expressed high levels of *BMP7*, whereas it was undetectable in the malignant B cells from two Diffuse large B-cell lymphoma (DLBCL) patients.

Analysis of *BMP6* and *BMP*7 expression levels across non-Hodgkin’s lymphoma (NHL) in an independent data set [16], showed good correlation with the RT-PCR data of purified malignant B cells (Compare Figure 1C and 1D). *BMP7* was highly expressed in FL as well as in the normal counterparts, but was expressed at low levels in most DLBCL (Figure 1D). Expression of *BMP7* in lymphoma cell lines was further confirmed at the protein level (Figure 1E and 1F), but did not correlate well with mRNA levels. Altogether, the expression of *BMP6* and *BMP7* in normal and malignant B cells suggests the possibility for autocrine growth regulation.

### B-cell Lymphoma Cells can Escape BMP-induced Inhibition of Cell Growth

**Figure 2:**
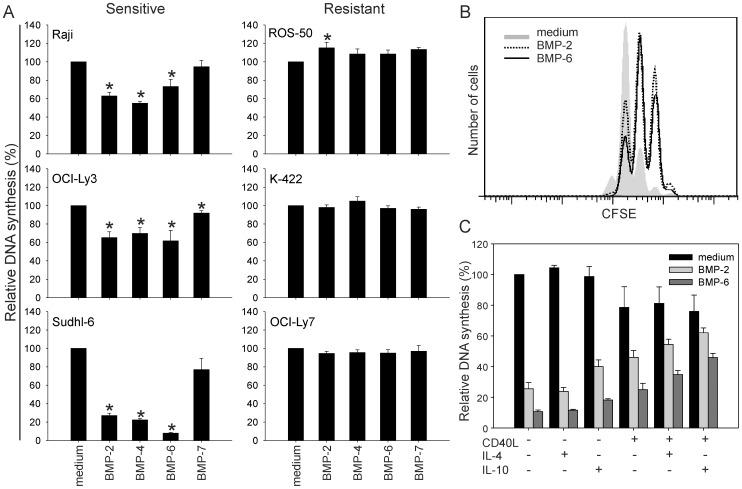
Effects of BMPs on proliferation of B-cell lymphoma cell lines. (A and C) Lymphoma cell lines were stimulated with various BMPs alone (A; *n* = 6, *p<0.05) or in the presence of interleukins and CD40L (C; Sudhl-6 cells, *n* ≥2) for three days before measuring ^3^H-thymidine incorporation. Results are normalized to unstimulated control in each experiment. (B) Tracking of cell division by CFSE labeling of Sudhl-6 cells cultured with or without BMP-2 or BMP-6 for three days. One representative of four independent experiments is shown.

As malignant B cells expressed *BMP6* and *BMP7* (Figure 1), we next studied the effects of exogenously added BMPs in different B-cell lymphoma cell lines. In addition to BMP-6 and BMP-7, we also included BMP-2 and BMP-4, since these BMPs constitute another subgroup of BMPs. BMP-2, -4 and -6 induced more than 30% inhibition of DNA synthesis in three cell lines (Raji, Sudhl-6, OCI-Ly3) of which Sudhl-6 was most affected (Figure 2A). These were defined as BMP sensitive. In contrast, three other cell lines (ROS-50, K-422, OCI-Ly7) were completely resistant to BMP-induced inhibition of DNA synthesis. Four cell lines (Bjab, Ramos, Sudhl-4, OCI-Ly10) showed intermediate sensitivity with less than 30% inhibition for any BMP tested (Figure S2). Interestingly, sensitivity to BMP-7 was low in all cell lines, with less than 20% inhibition of DNA synthesis (Figure 2A and Figure S2). In sensitive Sudhl-6 cells, CFSE tracking of cell division confirmed that proliferation was inhibited by BMP-2 and BMP-6 (Figure 2B). Induction of cell death was less prominent, except for Sudhl-6 cells (Table S1 and Table S2). Altogether, B-cell lymphoma cell lines had variable sensitivity to BMP-2-, -4- and -6-induced growth inhibition, but they were all resistant to BMP-7.

### Cytokines can Counteract the Inhibitory Effects of Exogenous BMP-6 in Sensitive Cells

The lymph node microenvironment normally contains many cytokines including IL-2, IL-4 and IL-10, mainly produced by activated T cells which also express membrane-bound CD40L [17]. Thus, we next tested if these B-cell stimulators could counteract the inhibitory effects of exogenous BMP-2 and BMP-6 in sensitive Sudhl-6 cells. IL-10 and CD40L partly abolished the effects of BMP-2 and BMP-6 by themselves, but the combination of these was more potent and increased DNA synthesis in BMP-6-stimulated cells (Figure 2C). In contrast, IL-4 or IL-2 did not counteract the effects of BMPs (Figure 2C and data not shown). Taken together, IL-10 and CD40L could partly counteract the BMP-induced growth suppression in sensitive Sudhl-6 cells.

### Sensitive and Resistant Lymphoma Cells Express BMP Receptors

**Figure 3:**
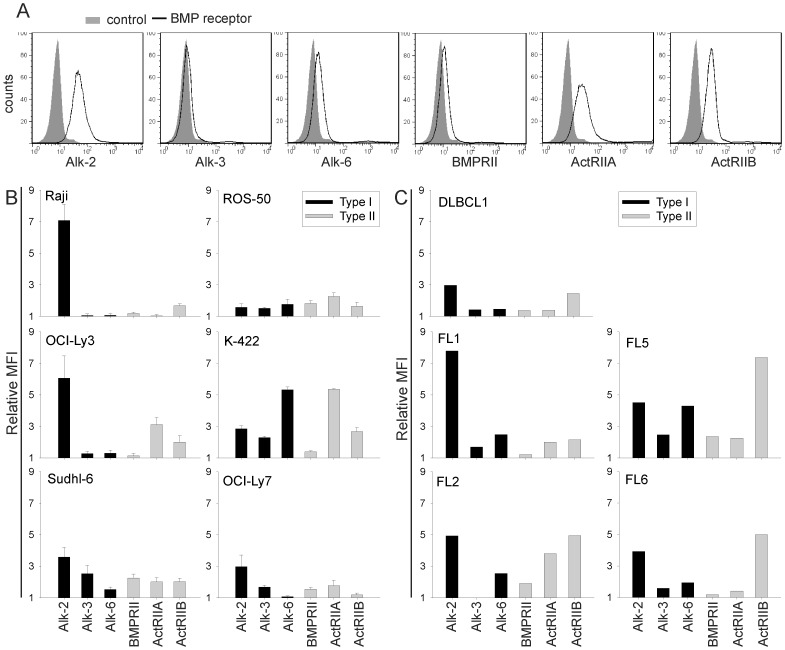
BMP-sensitive and -resistant lymphoma cells express BMP receptors. (A) BMP receptor expression in OCI-Ly3. (B-C) Relative receptor expression in lymphoma cell lines (B; means ± SEM, *n* = 3) and CD20^+^CD3^−^Igκ/Igλ^+^ malignant B cells from lymphoma patient samples (C). Values represent median fluorescent intensity (MFI) of each BMP receptor relative to the MFI of the isotype control.

Next, we focused on how some lymphoma cells could escape BMP-induced growth suppression by comparing BMP-induced signal transduction in sensitive and resistant cell lines. Expression of BMP receptors are reduced in several types of cancer and this could be a mechanism to evade BMP-induced suppression of proliferation [14], [18]–[20]. We used FACS analysis to determine the expression of BMP receptors. The sensitive cell line OCI-Ly3 is shown as an example and expressed high levels of activin receptor-like kinase (Alk) 2, activin receptor type II (ActRII) A and ActRIIB, and low levels of the other receptors (Figure 3A). All resistant cell lines expressed at least one type I and one type II receptor at comparable levels to sensitive cell lines (Figure 3B). In addition, the resistant cell line K-422 expressed high levels of receptors compared to sensitive cell lines.

We also included tumor samples from lymphoma patients, and malignant B cells from all patients expressed Alk-2 and ActRIIB (Figure 3C). Most of them also expressed Alk-6 and ActRIIA. Furthermore, the expression of the various BMP receptors was not different from the normal B cells present in the same sample (Table 1). These results indicate that downregulation of receptors is not a common mechanism for loss of BMP sensitivity in lymphomas.

**Table 1:** BMP receptor expression in tumor B cells and in normal infiltrating B cells from FL patient lymph node specimens.

		Relative MFI					
Patient	B-cell subset	Alk-2	Alk-3	Alk-6	BMPRII	ActRIIA	ActRIIB
FL1	Tumor	7.8	1.7	2.5	1.2	2.0	3.1
	Normal	2.9	1.9	2.6	1.5	1.9	2.2
FL2	Tumor	4.9	nd^a^	2.5	1.9	3.8	5.0
	Normal	3.6	nd^a^	1.6	1.4	1.5	6.3
FL5	Tumor	4.5	2.5	4.3	2.4	2.3	7.4
	Normal	1.5	1.1	1.2	1.0	1.0	5.0
FL6	Tumor	3.9	1.6	1.9	1.2	1.4	5.0
	Normal	2.3	1.3	1.9	0.9	1.1	6.6

a nd = not determined.

### BMP-induced Growth Suppression Correlates with Activation of Smad1/5/8

**Figure 4:**
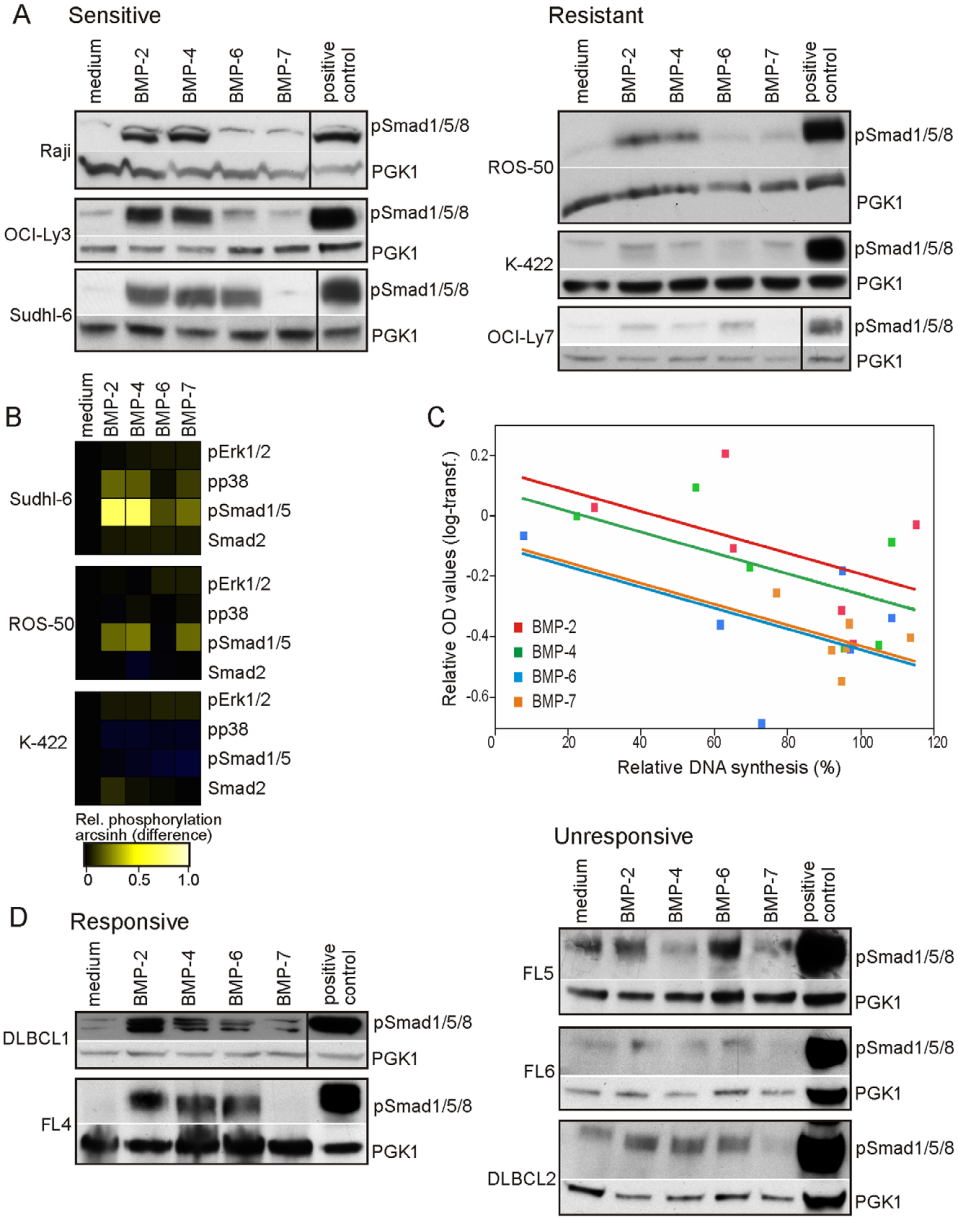
Reduced phosphorylation of Smad1/5/8 in resistant lymphoma cells. (A) Lymphoma cell lines were stimulated with BMPs for one hour and analyzed for the expression of pSmad1/5/8 by Western blotting. (B) BMP-induced phosphorylation of Smad1/5 and non-Smad pathways were determined by phospho-flow cytometry in lymphoma cell lines treated with or without BMPs for one hour. Shown is one representative of three independent experiments. (C) BMP-induced phosphorylation of Smad1/5/8 was quantified by densitometric analysis and normalized to the positive control, and mean OD-values (*n* = 3) are plotted against mean values for relative DNA synthesis for the corresponding BMP (from Figure 2A) for each cell line. The parallel lines are based on analysis of covariance; *p* = 0.015. (D) Tumor samples from five different lymphoma patients were treated with BMPs for one hour and analyzed for the expression of pSmad1/5/8. Anti-PGK1 was used as loading control and vertical lines indicate cutting of gel.

Next, we tested if resistance to BMP could be due to changes upstream or downstream of Smad1/5/8 activation. To optimize conditions, time-course experiments were performed in sensitive Sudhl-6 cells (Figure S3A), and a one-hour incubation period was selected for studies of BMP-induced signaling. Specificity of the BMPs was shown using Dorsomorphin, a selective inhibitor of BMPs [21], which completely abolished BMP-induced phosphorylation of Smad1/5/8 and growth-inhibitory effects (Figure S3B and C). Two out of three resistant cell lines showed very low or no phosphorylation in response to any of the BMPs tested compared to the positive control, which was the same for all Western blots (Figure 4A). In resistant ROS-50 cells, BMP-2 and BMP-4 induced activation of Smad1/5/8, but note that BMP-2 significantly increased the DNA synthesis in these cells (Figure 2A). As expected, BMP-2 and BMP-4 induced strong phosphorylation of Smad1/5/8 in the three sensitive cell lines compared to the positive control (Figure 4A). BMP-7 did not induce phosphorylation of Smad1/5/8, which is in agreement with no/limited inhibitory effects of this BMP. However, BMP-7 was active as it showed the same potency as BMP-2, BMP-4 and BMP-6 in inducing phoshorylation of Smad1/5/8 in peripheral blood B cells from healthy donors (Figure S4). The difference in BMP-2- and BMP-4-induced phosphorylation of Smad1/5 in Sudhl-6, ROS-50 and K-422 cells was confirmed by phospho-flow cytometry (Figure 4B). Activation of non-Smad signaling pathways, primarily p38 MAPK, was only seen in Sudhl-6 cells (Figure 4B).

To test if BMP-induced phosphorylation of Smad1/5/8 correlated with the BMPs’ antiproliferative capacity, we quantified the levels of pSmad1/5/8 in the Western blots. To compare different Western blots, we normalized the levels of pSmad1/5/8 to the positive control, which was the same for all the blots. The correlation between BMP-induced phosphorylation of Smad1/5/8 and suppression of DNA synthesis in the various lymphoma cell lines was significant when all the different BMPs were combined (*p* = 0.015, adjusted R-square = 40%; Figure 4C). Similar to the variable levels of BMP-induced phosphorylation of R-Smads in cell lines, analysis of BMP-induced phosphorylation in five lymphoma patient samples showed that lymphoma cells from two patients were responsive whereas the three others were unresponsive (Figure 4D). Furthermore, BMP-7 did not activate Smad1/5/8 in any of the patient samples. Note that the BMP receptor levels in the unresponsive samples were not different from those which showed BMP-induced phosphorylation of Smad1/5/8 (Figure 3C and 4D). As this is similar to what we observed for lymphoma cell lines, we expect that primary lymphoma cells can escape the negative influence of BMPs also *in vivo*. Collectively, BMP-induced phosphorylation of Smad1/5/8 correlated with the functional effects of BMPs in lymphoma cell lines, suggesting that BMP-resistance mechanisms in lymphoma are upstream of R-Smad activation.

### Overexpression of Smad7 in Sensitive Cell Lines Renders them Resistant to BMP

**Figure 5:**
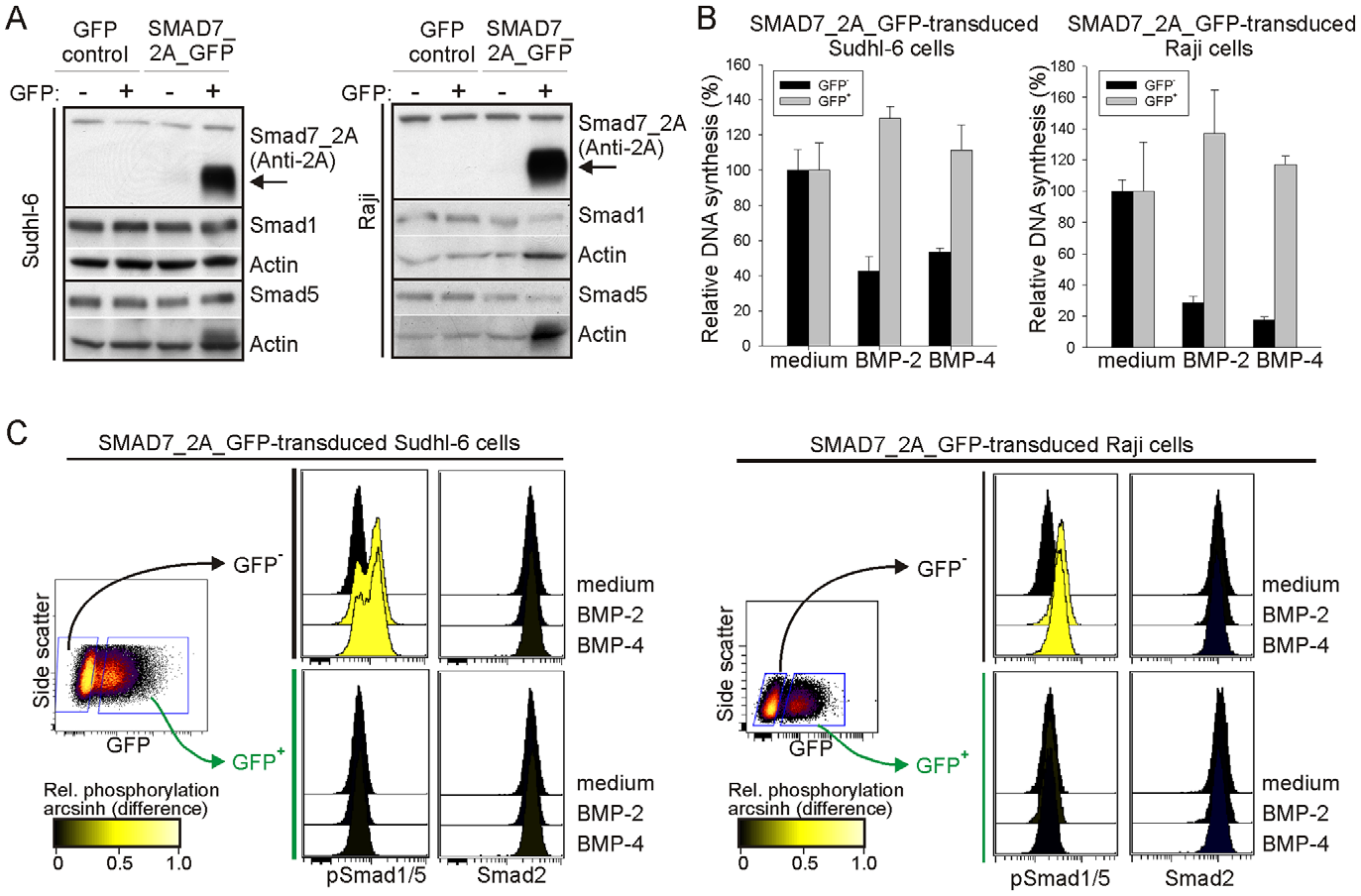
Overexpression of Smad7 in sensitive lymphoma cells renders them resistant to BMP-induced growth inhibition. Sudhl-6 and Raji cells were retrovirally transduced with GFP control vector or SMAD7_2A_GFP vector, and FACS sorted into GFP^−^ or GFP^+^ cells. Smad1, Smad5 and 2A-tagged Smad7 expression was measured by Western blotting, and anti-actin was used as loading control. (B) SMAD7_2A_GFP-transduced cells were treated with or without BMP-2 or BMP-4 for three days before ^3^H-thymidine incorporation was measured. Results are normalized to the unstimulated control (mean ± SD of triplicate wells). The experiments have been reproduced. (C) BMP-induced signaling was measured by treating retroviral transduced cells with or without BMP-2 or BMP-4 for one hour, followed by detection of GFP, pSmad1/5 and Smad2 by phospho-flow cytometry. The experiments have been reproduced.

Overexpression of inhibitory Smads is a potential mechanism of resistance, as these can inhibit BMP signaling in multiple ways, including binding to BMP type I receptors and preventing activation of R-Smads [22]. To test if upregulation of Smad7 in sensitive cell lines could render them less sensitive to BMPs, we used retroviral transduction to stably overexpress Smad7 in the BMP-sensitive cell lines Sudhl-6 and Raji. We used a bicistronic vector where *SMAD7* is linked to *GFP* (see materials and methods), and Smad7^+^ cells could therefore be indirectly identified based on the expression of GFP. By using FACS sorting, we obtained purified GFP^+^ and GFP^-^ cell subsets, and GFP^+^ cells transduced with SMAD7_2A_GFP vector had high expression of 2A-tagged Smad7 (Figure 5A). Furthermore, *SMAD7* mRNA was overexpressed in GFP^+^ Sudhl-6 cells transduced with the SMAD7_2A_GFP vector (Figure S5). Ectopic expression of Smad7 did not alter the expression of R-Smads (Figure 5A) or BMP receptors (Figure S6).

To determine the functional outcome of Smad7 overexpression, we tested the effects of BMP-2 and BMP-4 on DNA synthesis in GFP^−^ and GFP^+^ FACS-sorted cell populations from SMAD7_2A_GFP-transduced Sudhl-6 and Raji cells. Interestingly, overexpression of Smad7 altered the cells’ sensitivity to BMPs, as BMP-2 and BMP-4 inhibited DNA synthesis in the GFP^−^ population, in contrast to no inhibition in the GFP^+^ population of SMAD7_2A_GFP-transduced cells (Figure 5B). Transduction with the GFP control vector did not affect the sensitivity to BMPs (Figure S7A). BMP signaling was also measured by phospho-flow cytometry in SMAD7_2A_GFP-transduced Sudhl-6 and Raji cells. In agreement with the results from the DNA-synthesis assay, BMP-2 and BMP-4 induced phosphorylation of Smad1/5 in GFP^−^ cells, but not in the GFP^+^ cells overexpressing Smad7 (Figure 5C). In cells transduced with GFP control vector, BMP-2 and BMP-4 induced the same levels of phosphorylated Smad1/5 in GFP^−^ and GFP^+^ cells (Figure S7B), showing that expression of GFP did not affect BMP signaling. In SMAD7_2A_GFP-transduced Sudhl-6 cells, we saw some expression of Smad7 also in GFP^−^ cells (Figure S5) which can explain the slightly lower sensitivity to BMP-2 seen in the GFP^−^ population (compare Figure 5B and C with Figure S7). Taken together, these results show that overexpression of Smad7 is sufficient for lymphoma cells to become resistant to BMPs.

### Downregulation of R-Smads and Upregulation of Inhibitory Smads are Potential Mechanisms for Loss of BMP Responsiveness

**Figure 6:**
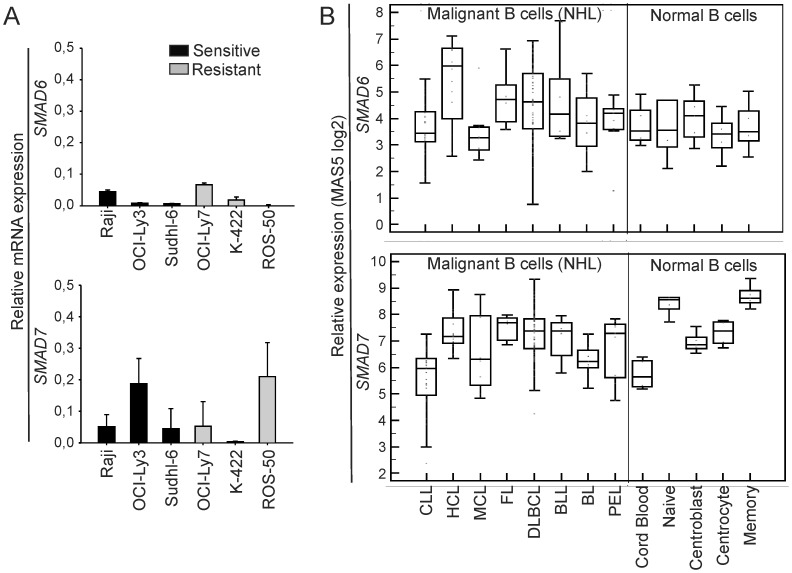
Expression of inhibitory *SMAD*s in lymphoma cells. (A) Real-time RT-PCR analysis of *SMAD6* and *SMAD7* expression in lymphoma cell lines, relative to their expression in the osteogenic sarcoma cell line Saos-2 (*SMAD6*; means ± SD, *n* = 2) or human fetal brain tissue (*SMAD7*; means ± SD, *n* = 5). (B) Relative expression of inhibitory *SMAD*s across NHLs (left of line) and in normal B-cell populations (right of line) obtained from a gene expression data set from Basso et al. [16].

Using real-time RT-PCR, we next investigated if resistant cells expressed higher levels of inhibitory *SMAD*s, and found that *SMAD6* and *SMAD7* mRNA levels varied among both the sensitive and the resistant cell lines (Figure 6A). Resistant ROS-50 cells had the highest level of *SMAD7* mRNA. Unfortunately, despite testing most commercially available anti-human Smad7 antibodies, we were not able to reliably detect Smad7 at the protein level. Retroviral transduction with a dominant negative Smad7 did not restore sensitivity in ROS-50 (Figure S8), and therefore, high levels of Smad7 seem not to be the mechanism for the BMP resistance seen in this cell line. However, gene expression profiling data across NHL showed that Chronic lymphocytic leukemia (CLL) and Mantle cell lymphoma (MCL) had lower levels of *SMAD6* and *SMAD7*, compared to the other B-cell malignancies (Figure 6B and Figure S9). Furthermore, the expression of inhibitory *SMAD*s also varied within each entity. This variability in expression levels of inhibitory *SMAD*s seen in cell lines and in primary patient samples, together with our finding that upregulation of Smad7 was sufficient to transform BMP-sensitive cells into resistant cells, suggest that overexpression of inhibitory Smads can be a potential mechanism for BMP resistance in some lymphoma cells.

Downregulation of Smad4 represents another possibility for development of BMP resistance. Although the protein level of Smad4 varied between lymphoma cell lines (Figure S10A), it did not correlate with sensitivity to BMPs. Of the resistant cell lines, only OCI-Ly7 had low expression of Smad4, but the level was not lower than in BMP-sensitive Sudhl-6 cells. Interrogation of *SMAD4* gene expression across different NHLs showed that *SMAD4* levels were comparable, also to normal counterparts (Figure S10B).

**Figure 7:**
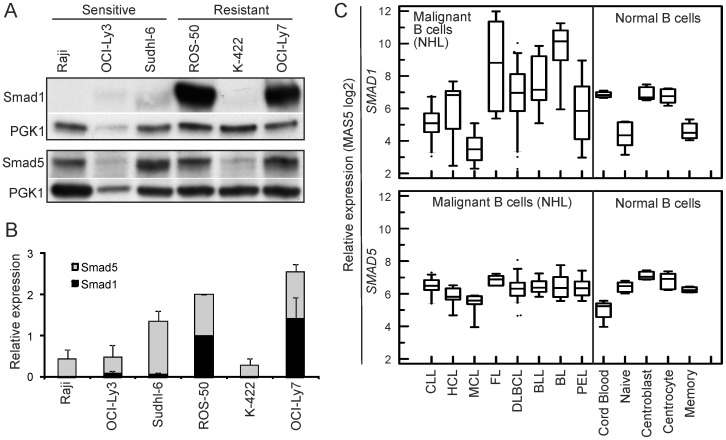
Expression of R-Smads in lymphoma cells. (A–B) Smad1 and Smad5 expression was measured by Western blotting in cell lines. Anti-PGK1 was used as loading control. One representative experiment (A) and mean values of optical density from densitometric measurements (B; ± SEM, *n* = 3) are shown. (C) Relative expression of *SMAD1* and *SMAD5* across NHLs in data set from Basso et al. [16].

The expression of Smad1 and Smad5 also varied between the cell lines (Figure 7A and B). As the resistant K-422 cells expressed lower levels of Smad1 and Smad5 than the other cell lines, this suggests downregulation of R-Smads as a possible mechanism for loss of BMP responsiveness in this cell line (Figure 7A and B). Interrogation of *SMAD1* gene expression across different NHLs showed that *SMAD1* varied across different subtypes, with highest levels in Burkitt lymphoma (BL), and lowest in MCL (Figure 7C). In contrast, only small variations in *SMAD5* levels were observed (Figure 7C). Altogether, altered expression of Smad proteins represents possible mechanisms for resistance to BMPs in B-cell lymphoma.

## Discussion

BMPs are known to inhibit proliferation and induce apoptosis in many cell types, including B cells [7], [8]. In cancer, alterations have been found in several components of the BMP signaling pathway, leading to BMP resistance. In this study, we have shown that B-cell lymphoma cells as well as normal germinal-center B cells express *BMP* mRNA, most frequently *BMP7*. Strikingly, we observed that all lymphoma cells were resistant to this BMP, whereas their sensitivity to BMP-2, -4 and -6 varied from highly sensitive to completely resistant. Our data suggest that more than one mechanism is involved in the resistance to BMPs, including downregulation of R-Smads and upregulation of inhibitory Smads.

We have previously shown that BMP-7 inhibits the growth of human, mature B cells by potently inducing apoptosis, whereas BMP-2, -4 and -6 had less effect [8]. In this study, lymphoma cell lines as well as malignant B cells from lymphoma patients were resistant to BMP-7. Combined with expression of *BMP7* in both normal and malignant B cells, this suggests that lymphoma cells can escape autocrine growth-inhibitory effects of BMP-7. Similarly, aggressive metastatic melanoma cells have been shown to be resistant to autocrine growth-inhibitory effects of BMP-7 [23]. Furthermore, *BMP7* expression correlated with tumor progression, as aggressive melanomas expressed abundant *BMP7* mRNA, but primary melanomas did not [23]. The relationship between BMP-7 and survival of lymphoma patients has not been studied, but expression of *BMP6* has previously been associated with prognosis in patients with hematological malignancies. In multiple myeloma patients, Seckinger et al. showed that high *BMP6* expression correlated with increased overall survival [24]. In DLBCL, hypermethylation of the *BMP6* promoter which correlated with lack of BMP-6 expression, was associated with decreased survival [15]. However, a study by Rosenwald et al., where *BMP6* increased the prognostic value of a gene expression signature in DLBCL, showed that *BMP6* expression was associated with poor outcome [25]. The finding that BMPs have a role in hematological malignancies is further strengthened as Grčević et al. [26] showed that expression of *BMP4* and *BMP6* was significantly higher in bone-marrow samples from multiple myeloma patients than from healthy controls.

Both sensitivity and resistance to BMPs have been reported, which is in line with the variation we observed in sensitivity to BMPs. Cancer cells from solid tumors were resistant to BMPs [14], [18], [20], whereas BMPs induced apoptosis and inhibited proliferation in multiple myeloma [27]–[29]. In ROS-50 cells, we found that BMP-2 induced DNA synthesis, suggesting rewired signaling and induction of target genes different from those normally induced when BMPs have antiproliferative effects. Others have also shown that BMP-2 can induce tumor growth under certain conditions [30], [31].

Downregulation of BMP receptors has been shown in cancer, and lost sensitivity can be restored by exogenous expression of BMPRII [14], [18], [20]. In a lymphoma cell line, resistance to TGF-β correlated with epigenetic silencing of TGF-β receptor II [32]. However, we did not detect loss of BMP receptors in lymphoma cell lines or lymphoma patient samples. Furthermore, the BMP receptor expression in lymphoma cells did not differ from the non-malignant B cells in patient samples. Thus, escape from BMP-induced growth inhibition in B-cell lymphomas seems not to be mediated via downregulation of BMP receptors. However, we cannot rule out the possibility for mutations in the intracellular domains of the receptors, preventing activation of R-Smads.

Although we found a statistical significant relationship between BMP-induced Smad1/5/8 activation and growth inhibition, BMPs induced Smad1/5/8 phosphorylation in one resistant cell line. This implies that the mechanism of resistance also can be found downstream of Smad1/5/8 phosphorylation, and that lymphoma cells can develop different ways to escape the negative influence of BMPs. Smad4 was originally identified as a tumor suppressor and deletions or mutations in *SMAD4* is common in solid cancers, including 50% of all pancreatic cancers [10]. However, mutations in *SMAD* genes are rare in hematopoietic tumors [33]. All lymphoma cell lines in this study expressed Smad4 although at different levels, and *SMAD4* mRNA had similar expression levels across different NHLs. In contrast, downregulation of R-Smads are more likely to contribute to BMP resistance. R-Smads have previously been shown to have a tumor-suppressive role, as gonade-specific deletion of *SMAD1/5* induces ovarian or testicular cancer in mice [34]. It has been suggested that microRNA-155 has a role in lymphomagenesis as it is highly expressed in some lymphomas [35], [36]. MicroRNA-155 expression can lead to limited cytostatic effect of BMPs through direct suppression of *SMAD5* [37], and has been shown to be overexpressed in the aggressive ABC subtype of DLBCL [35], [37], [38]. Hence, downregulation of Smad5 could represent a common way to escape BMP-induced growth suppression.

Upregulation of inhibitory Smads represents another possible mechanism of BMP resistance. Others have shown that *SMAD6* and *SMAD7* are upregulated in pancreatic cancer [39], [40]. Furthermore, Smad6 and Smad7 correlate with poor prognosis in lung cancer [41] and gastric carcinomas [42]. Importantly, we found that stable overexpression of Smad7 in BMP-sensitive lymphoma cells transformed them into BMP-resistant cells, showing that upregulation of Smad7 is sufficient for cancer cells to escape the negative effects of BMPs. Smad6 and Smad7 inhibit BMP signaling in multiple ways, including competition of binding to activated receptors or Smad4 [22]. In addition, Smad7 can inhibit Smad signaling in the nucleus by disrupting the formation of functional Smad-DNA complexes [43].

Whereas we were able to show that increasing the expression of Smad7 in two highly BMP-sensitive cell lines was sufficient to transform them into completely BMP-resistant cells, we have not been able to identify the mechanism for resistance in the three *de novo* resistant cell lines. In addition to ruling out alteration in expression of BMP receptors, expression of antagonists was also not associated with BMP resistance in these cells. Antagonists are secreted factors that bind BMPs and prevent receptor binding. We measured the mRNA expression of various BMP antagonists in sensitive and resistant cell lines. However, low or no expression was observed for all antagonists analyzed (data not shown). Hence, upregulation of BMP antagonists seems not to be a common way for lymphomas to escape BMP-induced growth inhibition. In addition to antagonists, several co-receptors have been identified which also can modulate BMP sensitivity in cancer cells [44], but this was not investigated here. Furthermore, translocations and mutations cause overexpression of oncogenes like *BCL2*, *BCL6* and *MYC* in lymphoma cells. In multiple myeloma, it was recently shown that BMP-induced apoptosis required downregulation of *MYC*. However, myeloma cells with *MYC* translocations evaded BMP-induced apoptosis [45], adding further complexity to how cancer cells can evade negative influence of BMPs. Two of the resistant cell lines in the present study, K-422 and ROS-50, do not have *MYC* translocations, but instead have *BCL2* translocations. If *BCL2* also can modulate BMP sensitivity similar to *MYC*, will be a subject for further studies.

In addition to alterations in the BMP signaling pathway, cytokines normally present in the lymph node environment could influence the functional outcome of BMP signaling. We found that the inhibitory effects of BMPs could be reduced by adding CD40L and IL-10. Similarly, activation of CD40 was found to inhibit TGF-β effects through induction of Smad7 [46]. The mechanism for how IL-10/CD40L counteract the inhibitory effects of BMPs could be via their activation of MAPKs as this has been shown to have an antagonistic effect on BMP signaling in many developmental contexts (reviewed in Wu [47]). MAPKs can phosphorylate serines and threonines in the linker region of Smad1, which e.g. marks it for ubiquitinylation and subsequent degradation [48]. Furthermore, strong proliferative pathways might override the transcriptional responses to BMP signals, whereas the presence of constitutively active p38 MAPK might sensitize cells towards TGF-β family members [49]. Since CD40L and IL-10 are present in the lymphoma microenvironment, these proteins can contribute to BMP resistance.

In conclusion, we have shown that some B-cell lymphomas can escape BMP-induced antiproliferative effects and that this correlates with reduced Smad1/5/8 activation. A common mechanism for loss of BMP sensitivity is downregulation or loss of receptor expression, but this was not seen in lymphomas. Instead, we found that overexpression of Smad7 is sufficient for lymphoma cells to become resistant to BMPs. How loss of BMP sensitivity might influence lymphomagenesis warrants further investigations, for example by using xenograft models.

## Materials and Methods

### Human Samples

Tumor biopsies were obtained from patients with Follicular lymphoma (FL, *n* = 6) or *de novo* Diffuse large B-cell lymphoma (DLBCL, *n* = 2) at The Norwegian Radium Hospital between 1988 and 1993. Tonsils were obtained from children undergoing routine tonsillectomy and served as healthy controls. Biopsies and tonsils were obtained with written informed consent in accordance with the Declaration of Helsinki and the Regional Committees for Medical and Health Research Ethics, Region Eastern Norway, approved protocols REK# 2.2007.2949 and REK# 2010/1147a, respectively. Single-cell suspensions were prepared and stored in liquid nitrogen.

### Reagents and Antibodies

Biotinylated antibodies were from R&D Systems (MN, USA): anti-ActRIA, anti-BMPRIA, anti-BMPRIB, anti-BMPRII, anti-ActRIIA and anti-ActRIIB. Goat serum was purchased from Sigma-Aldrich (MO, USA) and Streptavidin-PE from Dako (Denmark). Anti-CD38-PC5 was from Beckman Coulter (CA, USA); anti-IgD-PE, anti-Igκ-APC, anti-Igλ-PE, anti-CD3-FITC and anti-CD10-FITC were from Dako and anti-CD3-Pacific Blue, anti-CD77-FITC, anti-CD20-PerCPCy5.5 was from Becton Dickinson (BD; CA, USA); anti-pSmad1/5/8, anti-Smad4 and anti-β-Tubulin were from Cell Signalling Technology (CST; MA, USA); anti-Smad1 was from Upstate (Millipore, MA, USA) and CST, anti-2A was from Millipore, anti-actin was from Santa Cruz Biotechnology (CA, USA) and anti-PGK1 was from Abcam. Anti-BMP-7 (clone M1-F8) was from AbD Serotec. Antibodies used for phospho-flow cytometry: anti-pErk-Alexa488 (T202/204) and anti-pp38-Alexa647 (T180/Y182) were from BD; anti-pSmad1/5 (Ser462/Ser465) and anti-Smad2 were from CST. See detailed list of antibodies in Table S3. Recombinant human (rh) BMP-2 (300 ng/ml), rhBMP-4 (50 ng/ml), rhBMP-6 (500 ng/ml), rhBMP-7 (400 ng/ml), rhTGF-β (10 ng/ml), rhIL-2 (10 ng/ml), rhIL-4 (40 ng/ml) and rhIL-10 (10 ng/ml) were purchased from R&D Systems. CD40L (0.25 µg/ml) were purchased from Alexis Biochemicals (Enzo Life Sciences, NY, USA). Dorsomorphin (Calbiochem, Merck, Germany) was used at 1.25 µM as this concentration inhibited the BMP-induced effects without being toxic as determined by dose-response experiments (data not shown). For detection of BMP-7 protein, the cells were treated with Brefeldin A (BD; diluted 1/1000 at final concentration) for 20 hours prior to preparation of cell lysate.

### Cell Culture

The cell lines Raji (purchased from DSMZ, Germany, in 2005), Sudhl-4, Sudhl-6 (gift from L.M. Staudt, Metabolism Branch, Center for Cancer Research, National Cancer Institute, National Institutes of Health, Bethesda, MD; in 2005), K-422, ROS-50 (gift from J. Delabie, Department of Pathology, Oslo University Hospital, Oslo, Norway; in 2006), Ramos and Bjab (from Steinar Funderud, Department of Immunology, Oslo University Hospital, Oslo, Norway) were cultured in RPMI 1640 (PAA Laboratories, Austria) supplemented with 10% fetal calf serum (PAA), penicillin and streptomycin. OCI-Ly7, OCI-Ly3 and OCI-Ly10 (all from L.M. Staudt; in 2006) were cultured in IMDM medium (Invitrogen, CA, USA) supplemented with 20% human plasma, 55 µM β-mercaptoethanol, penicillin and streptomycin. In all experiments, cells were cultured in serum-free X-VIVO15 medium (BioWhittaker, Switzerland). Sudhl-6 obtained from DSMZ (ACC 573) in September 2009 gave identical results to Sudhl-6 cells obtained from L. Staudt. The cell lines Sudhl-4, Sudhl-6, ROS-50, K-422 and Ramos were authenticated in February 2011 (STR DNA profiling by RT-PCR of 16 polymorphic markers).

### Flow Cytometry, Fluorescence-activated Cell Sorting (FACS) and Phospho-specific Flow Cytometry

All incubations with antibodies were performed in the dark at 4°C for 30 min and flow-cytometry analysis was performed on a FACSCalibur (BD) or LSR II (BD). Centrocytes and centroblasts were isolated from children undergoing routine tonsillectomy as described previously [50]. Tumor-cell suspensions from lymphoma patients were stained with antibodies and subjected to FACS sorting into malignant B cells (CD20^+^CD10^+^CD3^−^Igκ/Igλ^+^) or infiltrating T cells (CD20^−^CD10^−^CD3^+^). Retrovirally transduced cell lines were also subjected to FACS sorting to obtain GFP^−^ and GFP^+^ cells. Phospho-specific flow cytometry was performed as described previously [51], see details in Methods S1. Flow-cytometry data was analyzed using FlowJo or Cytobank software (www.cytobank.org).

### Western Blotting

Cells were lysed in SDS lysis buffer and separated in polyacrylamide gels (Thermo Scientific, IL, USA) as previously described [49]. Protein bands were visualized by the ECL or ECL Plus detection systems (GE Healthcare, NJ, USA). Densitometric analysis was performed by scanning hyperfilms on a GS-800 Calibrated Densitometer (BioRad, CA, USA), using Quantity One software (Bio-Rad). Quantification of BMP-7 in each sample was calculated as the sum of the two bands (uncleaved monomer + propeptide) and normalized to that sample’s level of β-tubulin, and then calculated relative to the expression in Raji cells. Quantification of phosphorylated Smad1/5/8 was calculated relative to the expression in the positive control, which was cell lysate prepared from Sudhl-6 cells treated with BMP-2 for one hour.

### Proliferation Assay and Cell-division Tracking

Cells were cultured in triplicates in 96-well round-bottom plates (20 000 cells/well in 200 µl) for 3 days and 20 µl of ^3^H-thymidine was added the 4 last hours of incubation. ^3^H-thymidine incorporation was measured as described previously [8].

For cell-division tracking, cells were labeled with 5 µM CFSE (Molecular Probes, OR, USA) for 10 min at 37°C, washed in PBS w/10% FCS and FACS sorted (50 channels around CFSE peak). Sorted cells were cultured with or without BMPs for 3 days (20 000 cells/well in 200 µl) before FACS analysis of CFSE intensity was performed on a FACS Canto (BD).

### Overexpression of Wild Type and Dominant Negative Smad7 by Retroviral Transduction

SMAD7_2A_GFP construct was designed *in silico* and synthesized *in vitro* by GeneArt (Invitrogen). The construct is a bicistronic gene where *SMAD7* (isoform 1) coding sequence (CDS) devoid of STOP codon is linked to *GFP* CDS via a *Picornavirus* 2A sequence (TNFSLLKQAGDVEENPG*P, where * represents 2A cleveage site). 2A peptides allow multiple proteins to be encoded as polyproteins, which then dissociate into component proteins upon translation by ribosomal skipping. Therefore, 2A-tagged Smad7 and GFP will be produced as separated proteins, but at almost equimolar amounts. SMAD7_2A_GFP construct was subcloned into a retroviral expression vector. Furthermore, a dominant negative *SMAD7* mutant (SMAD7DN) was constructed by a 25-amino acid deletion at the COOH terminus of Smad7 as previously described [52], and the SMAD7DN_2A_GFP construct was subcloned into the same retroviral expression vector. A retroviral vector expressing GFP (our collection) was used as a control vector. For viral particle preparation, Hek-Packaging cells were transfected and supernatants were harvested at 48 and 72 hours and pooled. Sudhl-6 and Raji cells (0.5 million) were transduced twice by spinoculation on Retronectin-coated (Takara, Japan) non-treated plates, for 1 hour at 900×g. Transduced cells were expanded for at least 10 days before they were used in experiments.

### Real-time RT-PCR and Gene Expression Data

RNA was isolated from cells using the Absolute RNA miniprep kit (Stratagene, CA, USA) following the manufacturer’s recommendations. cDNA was synthesized and analyzed by real-time RT-PCR using TaqMan technology (Applied Biosciences, CA, USA), and gene expression was quantified using the comparative C_T_ method as previously described [4]. *PGK1* was used as endogenous reference and gene expression in each sample was normalized to the level in fetal brain tissue (BioChain, CA, USA) or to the cell line Saos-2 as described in figure legends.

Data processing for the gene expression data set from Basso et al. [16], GSE2350, was done as previously described [53]. Briefly, Affymetrix raw data were normalized using MAS5.0 and Entrez gene version 12 probe summaries within a custom chip definition schema [54]. Results are shown as log_2_-transformed values, and included Chronic lymphoid leukemia (CLL, *n = *34); Hairy cell leukemia (HCL, *n = *16); Mantle cell lymphoma (MCL, *n = *8); FL (*n = *6); DLBCL (*n = *60); Burkitt like lymphoma (BLL, *n = *6); Burkitt lymphoma (BL, *n = *11) and Primary effusion lymphoma (PEL, *n = *9). The data set also included normal B-cell populations (*n = *5 for each population).

### Statistical Analysis

Statistical comparisons of groups were calculated using two-sided, paired Student’s t-tests. In Figure 2A the mean value of triplicates in each experiment was log-transformed before doing the t-test. The relation between BMP-induced phosphorylation of Smad1/5/8 and suppression of DNA synthesis was evaluated by analysis of covariance, using DNA synthesis and an indicator for the BMPs as factors in the analysis (JMP 7.0 software).

## Supporting Information

Figure S1 ***BMP4*** **mRNA expression in B-cell lymphoma cell lines.** *BMP4* mRNA expression was determined by real-time RT-PCR. Data are given relative to the expression of *BMP* in human fetal brain tissue. (Means ± SEM, *n* = 3).
(TIF)

Figure S2 **Inhibition of DNA synthesis in cell lines showing intermediate sensitivity to BMPs.** Lymphoma cell lines stimulated with or without BMPs for three days before ^3^H-thymidine incorporation was measured. Values are obtained by normalizing mean cpm for each BMP to the mean cpm for unstimulated control in each experiment. (Means ± SEM, *n* = 6–7) *p<0.05.
(TIF)

Figure S3 **BMP-induced phosphorylation of Smad1/5/8 is selectively blocked by Dorsomorphin in sensitive Sudhl-6 cells.** (A) Cells were cultured with BMP-2, -4 or -6 for different time periods, and analyzed for pSmad1/5/8 expression by Western blotting. (B) Cells stimulated with various BMPs or TGF-β with or without Dorsomorphin for one hour before cells were lysed to detect pSmad1/5/8 induction. (C) Cells were stimulated with or without various BMPs or TGF-β, in the presence or absence of Dorsomorphin for three days before ^3^H-thymidine incorporation was measured. (Mean cpm values ± SEM, *n* = 6 (*n = *2 for Dorsomorphin only)).
(TIF)

Figure S4 **BMP-7 induces pSmad1/5/8 to the same level as other BMPs in B cells from healthy donors.** CD19^+^ B cells were purified from peripheral blood from healthy donors and cultured with or without BMPs for one hour before pSmad1/5/8 expression was determined by Western blotting. Smad1 was used as loading control. One representative of four experiments is shown.
(TIF)

Figure S5 **Sudhl-6 cells transduced with SMAD7_2A_GFP vector highly express** ***SMAD7*** **mRNA.** Real-time RT-PCR analysis of *SMAD7* expression in Sudhl-6 cells which were retrovirally transduced with GFP control vector or SMAD7_2A_GFP vector and FACS sorted into GFP^−^ or GFP^+^ cells. Expression is shown relative to the expression in human fetal brain tissue, and one representative of two independent experiments is shown.
(TIF)

Figure S6 **Ectopic expression of Smad7 does not alter the expression of BMP receptors.** Relative BMP receptor expression in GFP^−^ and GFP^+^ Sudhl-6 cells transduced with SMAD7_2A_GFP vector (means ± SD, *n* = 2). Values represent median fluorescent intensity (MFI) of each BMP receptor relative to the MFI of the isotype control.
(TIF)

Figure S7 **Overexpression of GFP in sensitive cell lines does not alter their sensitivity to BMPs.** (A) Sudhl-6 and Raji cells were retrovirally transduced with GFP control vector, FACS sorted into GFP^−^ or GFP^+^ cells, and treated with or without BMP-2 and BMP-4 for three days before ^3^H-thymidine incorporation was measured. Results are normalized to unstimulated control in each experiment (mean ± SD of triplicate wells). The experiments have been reproduced. (B) BMP-induced signaling was measured by treating retrovirally transduced cells with or without BMP-2 or BMP-4 for one hour, followed by detection of GFP, pSmad1/5 and Smad2 by phospho-flow cytometry. The experiments have been reproduced.
(TIF)

Figure S8 **Expression of dominant negative Smad7 does not restore BMP sensitivity in ROS-50 cells.** (A) ROS-50 cells transduced with SMAD7DN_2A_GFP or GFP control vector were FACS sorted, and GFP^+^ cells were subjected to real-time RT PCR. A gene expression assay binding to the NH_2_ terminus of *SMAD7* (which also detects the *SMAD7DN* mutant) was used to measure the mRNA levels. Expression is shown relative to the expression in wild type ROS-50 cells and *PGK1* is used as endogenous control. (B) Western blotting was used to measure the expression of 2A-tagged Smad7DN in GFP^−^ and GFP^+^ ROS-50 cells, transduced with SMAD7DN_2A_GFP. Actin was used as loading control. (C) SMAD7DN_2A_GFP transduced ROS-50 cells were treated with or without BMP-2 or BMP-4 for one hour before they were fixed, permeabilized, stained with anti-pSmad1/5 or Smad2 and analyzed by flow cytometry (*n* = 2). Even though Smad7DN was highly expressed (shown in A and B), it did not alter the BMP sensitivity of ROS-50 cells. (D) To show that the Smad7DN is not a functional Smad7 protein, we transduced Sudhl-6 cells with the SMAD7DN_2A_GFP vector, in addition to the GFP control and SMAD7_2A_GFP vector (as shown in Figure 5). The cells were treated with or without BMP-2 or BMP-4 for one hour before they were fixed, permeabilized, stained with anti-pSmad1/5 or Smad2 and analyzed by flow cytometry. Results are shown for GFP^+^ populations only. Expression of Smad7DN did not make Sudhl-6 cells resistant as the ectopic overexpression of wild type Smad7 did.
(TIF)

Figure S9 ***SMAD7*** **mRNA is higher in FL and DLBCL as compared to CLL.** Relative expression of inhibitory *SMAD*s across NHLs in the data set from Alizadeh et al. [55], * *p*<0.0001 compared to CLL.
(TIF)

Figure S10 **Expression of Smad4.** (A) Smad4 expression in unstimulated cells, analyzed by Western blotting. Anti-PGK1 was used as loading control. Experiment was repeated once with similar results. (B) Relative expression of *SMAD4* across non-Hodgkin’s lymphoma (NHL; left of line) and in normal B-cell populations (right of line) obtained from the data set of Basso et al. [16].
(TIF)

Table S1 **Analysis of BMP-induced cell death in lymphoma cell lines.** Lymphoma cell lines were stimulated with or without BMPs for three days before PI-positive cells were detected by FACS analysis. Means ± SEM, n = 6–7, *p<0.05.
(DOC)

Table S2 **BMP-2 and -6 induce apoptosis in Sudhl-6.** Sudhl-6 cells were stimulated with BMPs for three days before apoptosis was quantified with TUNEL. Means ± SEM, n = 3.
(DOC)

Table S3 **Antibody specifications.**
(DOC)

Methods S1 (DOC)

## References

[1] ten Dijke P, Korchynskyi O, Valdimarsdottir G, Goumans MJ (2003) Controlling cell fate by bone morphogenetic protein receptors. Mol Cell Endocrinol 211: 105–113. 14656483 10.1016/j.mce.2003.09.016

[2] Bragdon B, Moseychuk O, Saldanha S, King D, Julian J, et al (2011) Bone Morphogenetic Proteins: A critical review. Cell Signal 23: 609–620. 20959140 10.1016/j.cellsig.2010.10.003

[3] Bhatia M, Bonnet D, Wu D, Murdoch B, Wrana J, et al (1999) Bone Morphogenetic Proteins Regulate the Developmental Program of Human Hematopoietic Stem Cells. J Exp Med 189: 1139–1148. 10190905 10.1084/jem.189.7.1139 PMC2193014

[4] Kersten C, Dosen G, Myklebust JH, Sivertsen EA, Hystad ME, et al (2006) BMP-6 inhibits human bone marrow B lymphopoiesis - Upregulation of Id1 and Id3. Exp Hematol 34: 72–81. 16413393 10.1016/j.exphem.2005.09.010

[5] Cejalvo T, Sacedón R, Hernández-López C, Diez B, Gutierrez-Frías C, et al (2007) Bone morphogenetic protein-2/4 signalling pathwaycomponents are expressed in the human thymus and inhibit early T-cell development. Immunology 121: 94–104. 17425602 10.1111/j.1365-2567.2007.02541.x PMC2265915

[6] Varas A, Martinez VG, Hernández-López C, Hidalgo L, Entrena A, et al (2009) Role of BMP signalling in peripheral CD4^+^ T cell proliferation. Inmunología 28: 125–130.

[7] Kersten C, Sivertsen EA, Hystad ME, Forfang L, Smeland EB, et al (2005) BMP-6 inhibits growth of mature human B cells; induction of Smad phosphorylation and upregulation of Id1. BMC Immunol 6: 9. 15877825 10.1186/1471-2172-6-9 PMC1134658

[8] Huse K, Bakkebø M, Oksvold MP, Forfang L, Hilden VI, et al (2011) Bone morphogenetic proteins inhibit CD40L/IL-21-induced Ig production in human B cells: differential effects of BMP-6 and BMP-7. Eur J Immunol 41: 3135–3145. 21898381 10.1002/eji.201141558

[9] Tsalavos S, Segklia K, Passa O, Petryk A, O’Connor MB, et al (2011) Involvement of Twisted Gastrulation in T Cell-Independent Plasma Cell Production. J Immunol 186: 6860–6870. 21572028 10.4049/jimmunol.1001833

[10] Seoane J (2006) Escaping from the TGFβ anti-proliferative control. Carcinogenesis 27: 2148–2156. 16698802 10.1093/carcin/bgl068

[11] Levy L, Hill CS (2002) Alterations in components of the TGF-β superfamily signaling pathways in human cancer. Cytokine Growth Factor Rev 17: 41–58. 10.1016/j.cytogfr.2005.09.009 16310402

[12] Kim IY, Kim SJ (2006) Role of bone morphogenetic proteins in transitional cell carcinoma cells. Cancer Lett 241: 118–123. 16500023 10.1016/j.canlet.2005.10.009

[13] Thawani JP, Wang AC, Than KD, Lin CY, Marca FL, et al (2010) Bone Morphogenetic Proteins and Cancer: Review of the Literature. Neurosurgery 66: 233–246. 20042986 10.1227/01.NEU.0000363722.42097.C2

[14] Kodach LL, Wiercinska E, de Miranda NFCC, Bleuming SA, Musler AR, et al (2008) The Bone Morphogenetic Protein Pathway Is Inactivated in the Majority of Sporadic Colorectal Cancers. Gastroenterology 134: 1332–1341. 18471510 10.1053/j.gastro.2008.02.059

[15] Daibata M, Nemoto Y, Bandobashi K, Kotani N, Kuroda M, et al (2007) Promoter Hypermethylation of the Bone Morphogenetic Protein-6 Gene in Malignant Lymphoma. Clin Cancer Res 13: 3528–3535. 17575215 10.1158/1078-0432.CCR-06-2766

[16] Basso K, Margolin AA, Stolovitzky G, Klein U, Dalla-Favera R, et al (2005) Reverse engineering of regulatory networks in human B cells. Nat Genet 37: 382–390. 15778709 10.1038/ng1532

[17] Choi YS (1997) Differentiation and Apoptosis of Human Germinal Center B-Lymphocytes. Immunol Res 16: 161–174. 9212362 10.1007/BF02786360

[18] Kim IY, Lee DH, Lee DK, Kim BC, Kim HT, et al (2003) Decreased Expression of Bone Morphogenetic Protein (BMP) Receptor Type II Correlates with Insensitivity to BMP-6 in Human Renal Cell Carcinoma Cells. Clin Cancer Res 9: 6046–6051. 14676131

[19] Kim IY, Lee DH, Lee DK, Ahn HJ, Kim MM, et al (2004) Loss of expression of bone morphogenetic protein receptor type II in human prostate cancer cells. Oncogene 23: 7651–7659. 15354178 10.1038/sj.onc.1207924

[20] Kim IY, Lee DH, Lee DK, Kim WJ, Kim MM, et al (2004) Restoration of Bone Morphogenetic Protein Receptor Type II Expression Leads to a Decreased Rate of Tumor Growth in Bladder Transitional Cell Carcinoma Cell Line TSU-Pr1. Cancer Res 64: 7355–7360. 15492256 10.1158/0008-5472.CAN-04-0154

[21] Yu PB, Hong CC, Sachidanandan C, Babitt JL, Deng DY, et al (2008) Dorsomorphin inhibits BMP signals required for embryogenesis and iron metabolism. Nat Chem Biol 4: 33–41. 18026094 10.1038/nchembio.2007.54 PMC2727650

[22] Sieber C, Kopf J, Hiepen C, Knaus P (2009) Recent advances in BMP receptor signaling. Cytokine Growth Factor Rev 20: 343–355. 19897402 10.1016/j.cytogfr.2009.10.007

[23] Hsu MY, Rovinsky S, Lai CY, Qasem S, Liu X, et al (2008) Aggressive melanoma cells escape from BMP7-mediated autocrine growth inhibition through coordinated Noggin upregulation. Lab Invest 88: 842–855. 18560367 10.1038/labinvest.2008.55 PMC2676927

[24] Seckinger A, Meissner T, Moreaux J, Goldschmidt H, Fuhler GM, et al (2009) Bone morphogenic protein 6: a member of a novel class of prognostic factors expressed by normal and malignant plasma cells inhibiting proliferation and angiogenesis. Oncogene 28: 3866–3879. 19718049 10.1038/onc.2009.257 PMC2844406

[25] Rosenwald A, Wright G, Chan WC, Connors JM, Campo E, et al (2002) The Use of Molecular Profiling to Predict Survival after Chemotherapy for Diffuse Large-B-Cell Lymphoma. N Engl J Med 346: 1937–1947. 12075054 10.1056/NEJMoa012914

[26] Grčević D, Kušec R, Kovačić N, Lukić A, Lukić IK, et al (2010) Bone morphogenetic proteins and receptors are over-expressed in bone-marrow cells of multiple myeloma patients and support myeloma cells by inducing ID genes. Leuk Res 34: 742–751. 19926132 10.1016/j.leukres.2009.10.016

[27] Ro TB, Holt RU, Brenne AT, Hjorth-Hansen H, Waage A, et al (2004) Bone morphogenetic protein-5, -6 and -7 inhibit growth and induce apoptosis in human myeloma cells. Oncogene 23: 3024–3032. 14691444 10.1038/sj.onc.1207386

[28] Hjertner O, Hjorth-Hansen H, Börset M, Seidel C, Waage A, et al (2001) Bone morphogenetic protein-4 inhibits proliferation and induces apoptosis of multiple myeloma cells. Blood 97: 516–522. 11154231 10.1182/blood.v97.2.516

[29] Kawamura C, Kizaki M, Ikeda Y (2002) Bone Morphogenetic Protein (BMP)-2 Induces Apoptosis in Human Myeloma Cells. Leuk Lymphoma 43: 635–639. 12002771 10.1080/10428190290012182

[30] Ide H, Yoshida T, Matsumoto N, Aoki K, Osada Y, et al (1997) Growth Regulation of Human Prostate Cancer Cells by Bone Morphogenetic Protein-2. Cancer Res 57: 5022–5027. 9371496

[31] Langenfeld EM, Kong Y, Langenfeld J (2005) Bone morphogenetic protein 2 stimulation of tumor growth involves the activation of Smad-1/5. Oncogene 25: 685–692. 10.1038/sj.onc.1209110 16247476

[32] Chen G, Ghosh P, Osawa H, Sasaki CY, Rezanka L, et al (2007) Resistance to TGF-β1 correlates with aberrant expression of TGF-β receptor II in human B-cell lymphoma cell lines. Blood 109: 5301–5307. 17339425 10.1182/blood-2006-06-032128 PMC1890833

[33] Kim SJ, Letterio J (2003) Transforming growth factor-β signaling in normal and malignant hematopoiesis. Leukemia 17: 1731–1737. 12970772 10.1038/sj.leu.2403069

[34] Pangas SA, Li X, Umans L, Zwijsen A, Huylebroeck D, et al (2008) Conditional Deletion of *Smad1* and *Smad5* in Somatic Cells of Male and Female Gonads Leads to Metastatic Tumor Development in Mice. Mol Cell Biol 28: 248–257. 17967875 10.1128/MCB.01404-07 PMC2223289

[35] Kluiver J, Poppema S, de Jong D, Blokzijl T, Harms G, et al (2005) BIC and miR-155 are highly expressed in Hodgkin, primary medistinal and diffuse large B cell lymphomas. J Pathol 207: 243–249. 16041695 10.1002/path.1825

[36] Roehle A, Hoefig KP, Repsilber D, Thorns C, Ziepert M, et al (2008) MicroRNA signatures characterize diffuse large B-cell lymhpomas and follicular lymphomas. Br J Haematol 142: 732–744. 18537969 10.1111/j.1365-2141.2008.07237.x

[37] Rai D, Kim SW, McKeller MR, Dahia PLM, Aguiar RCT (2010) Targeting of SMAD5 links microRNA-155 to the TGF-β pathway and lymphomagenesis. Proc Natl Acad Sci U S A 107: 3111–3116. 20133617 10.1073/pnas.0910667107 PMC2840369

[38] Rai D, Karanti S, Jung I, Dahia PLM, Aguiar RCT (2008) Coordinated expression of microRNA-155 and predicted target genes in diffuse large B-cell lymphoma. Cancer Genet Cytogenet 181: 8–15. 18262046 10.1016/j.cancergencyto.2007.10.008 PMC2276854

[39] Kleeff J, Maruyama H, Friess H, Büchler MW, Falb D, et al (1999) Smad6 Suppresses TGF-β-Induced Growth Inhibition in COLO-357 Pancreatic Cancer Cells and Is Overexpressed in Pancreatic Cancer. Biochem Biophys Res Commun 255: 268–273. 10049697 10.1006/bbrc.1999.0171

[40] Kleeff J, Ishiwata T, Maruyama H, Friess H, Truong P, et al (1999) The TGF-β signaling inhibitor Smad7 enhances tumorigenicity in pancreatic cancer. Oncogene 23: 5363–5372. 10.1038/sj.onc.1202909 10498890

[41] Jeon HS, Dracheva T, Yang SH, Meerzaman D, Fukuoka J, et al (2008) SMAD6 Contributes to Patient Survival in Non-Small Cell Lung Cancer and Its Knockdown Reestablishes TGF-β Homeostasis in Lung Cancer Cells. Cancer Res 68: 9686–9692. 19047146 10.1158/0008-5472.CAN-08-1083 PMC3617041

[42] Kim YH, Lee HS, Lee HJ, Hur K, Kim WH, et al (2004) Prognostic significance of the expression of Smad4 and Smad7 in human gastric carcinomas. Ann Oncol 15: 574–580. 15033661 10.1093/annonc/mdh131

[43] Zhang S, Fei T, Zhang L, Zhang R, Chen F, et al (2007) Smad7 Antagonizes Transforming Growth Factor β Signaling in the Nucleus by Interfering with Functional Smad-DNA Complex Formation. Mol Cell Biol 27: 4488–4499. 17438144 10.1128/MCB.01636-06 PMC1900056

[44] Bernabeu C, Lopez-Novoa JM, Quintanilla M (2009) The emerging role of TGF-β superfamily coreceptors in cancer. Biochim Biophys Acta 1792: 954–973. 19607914 10.1016/j.bbadis.2009.07.003

[45] Holien T, Våtsveen TK, Hella H, Rampa C, Brede G, et al (2011) Bone morphogenetic proteins induce apoptosis in multiple myeloma cells by Smad-dependent repression of MYC. Leukemia 26: 1073–1080. 21941367 10.1038/leu.2011.263

[46] Patil S, Wildey GM, Brown TL, Choy L, Derynck R, et al (2000) Smad7 Is Induced by CD40 and Protects WEHI 231 B-lymphocytes from Transforming Growth Factor-β-induced Growth Inhibition and Apoptosis. J Biol Chem 275: 38363–38370. 10995749 10.1074/jbc.M004861200

[47] Wu MY, Hill CS (2009) TGF-β Superfamily Signaling in Embryonic Development and Homeostasis. Dev Cell 16: 329–343. 19289080 10.1016/j.devcel.2009.02.012

[48] Sapkota G, Alarcon C, Spagnoli FM, Brivanlou AH, Massague J (2007) Balancing BMP Signaling through Integrated Inputs into the Smad1 Linker. Mol Cell 25: 441–454. 17289590 10.1016/j.molcel.2007.01.006

[49] Bakkebø M, Huse K, Hilden V, Smeland E, Oksvold M (2010) TGF-β-induced growth inhibition in B-cell lymphoma correlates with Smad1/5 signalling and constitutively active p38 MAPK. BMC Immunol 11: 57. 21092277 10.1186/1471-2172-11-57 PMC3006362

[50] Torlakovic E, Malecka A, Myklebust JH, Tierens A, Aashein HC, et al (2005) PU.1 protein expression has a positive linear association with protein expression of germinal centre B cell genes including *BCL-6*, *CD10*, *CD20* and *CD22*: identification of PU.1 putative binding sites in the *BCL-6* promotor. J Pathol 206: 312–319. 15892171 10.1002/path.1777

[51] Irish JM, Myklebust JH, Alizadeh AA, Houot R, Sharman JP, et al (2010) B-cell signaling networks reveal a negative prognostic human lymphoma cell subset that emerges during tumor progression. Proc Natl Acad Sci U S A 107: 12747–12754. 20543139 10.1073/pnas.1002057107 PMC2919949

[52] Huang M, Sharma S, Zhu LX, Keane MP, Luo J, et al (2002) IL-7 inhibits fibroblast TGF-β production and signaling in pulmonary fibrosis. J Clin Invest 109: 931–937. 11927620 10.1172/JCI14685 PMC150933

[53] Gentles AJ, Alizadeh AA, Lee SI, Myklebust JH, Shachaf CM, et al (2009) A pluripotency signature predicts histologic transformation and influences survival in follicular lymphoma patients. Blood 114: 3158–3166. 19636063 10.1182/blood-2009-02-202465 PMC2759646

[54] Dai M, Wang P, Boyd AD, Kostov G, Athey B, et al (2005) Evolving gene/transcript definitions significantly alter the interpretation of GeneChip data. Nucleic Acids Res 33: e175. 16284200 10.1093/nar/gni179 PMC1283542

[55] Alizadeh AA, Eisen MB, Davis RE, Ma C, Lossos IS, et al (2000) Distinct types of diffuse large B-cell lymphoma identified by gene expression profiling. Nature 403: 503–511. 10676951 10.1038/35000501

