# Supplementary figures and images for: Role of Smad Proteins in Resistance to BMP-Induced Growth Inhibition in B-Cell Lymphoma
Source: PLoS One. 2012 Oct 1;7(10):e46117. doi: 10.1371/journal.pone.0046117 (PMC3462182; doi:10.1371/journal.pone.0046117)

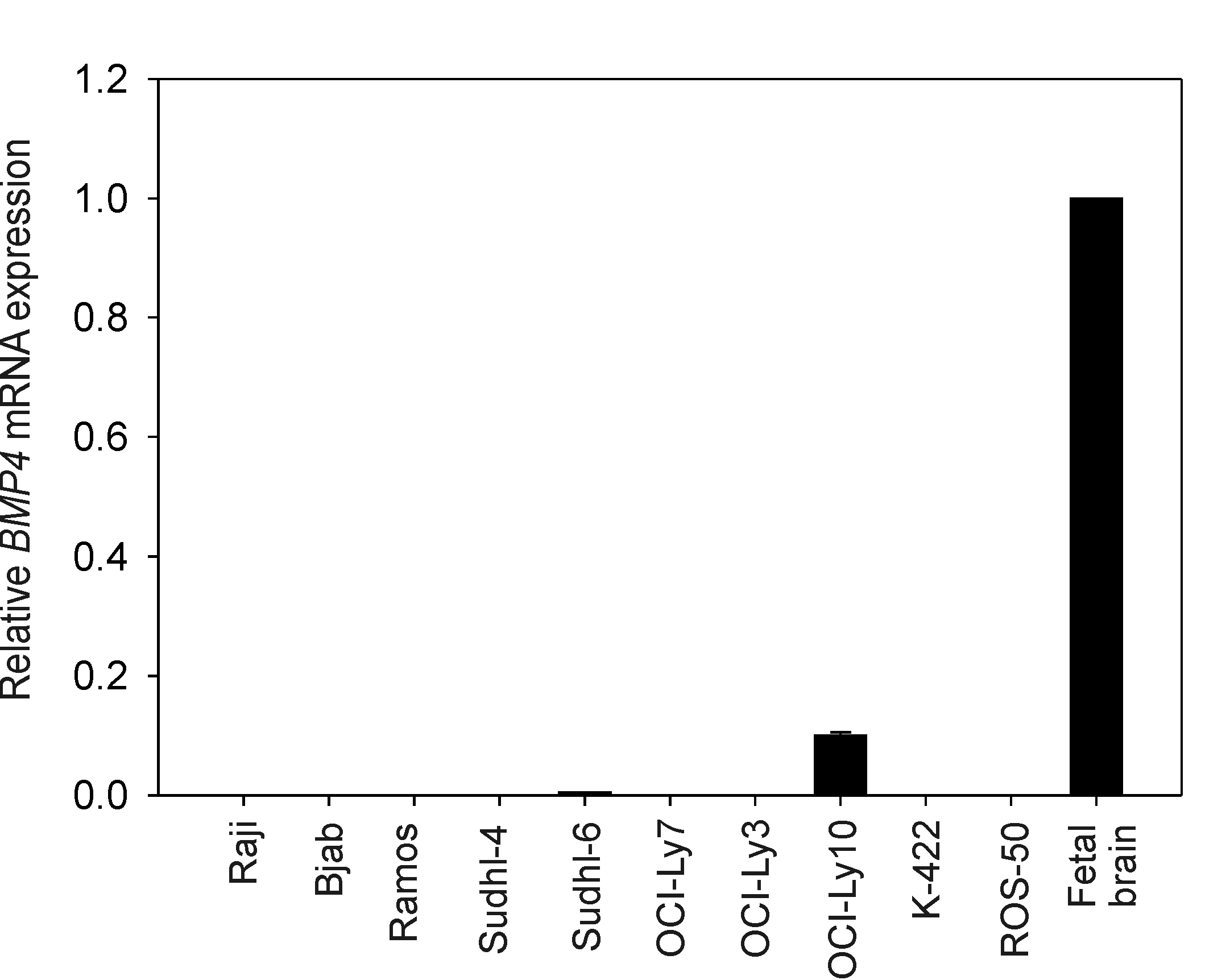

Supplement: Figure S1 — BMP4 mRNA expression in B-cell lymphoma cell lines. BMP4 mRNA expression was determined by real-time RT-PCR. Data are given relative to the expression of BMP in human fetal brain tissue. (Means ± SEM, n = 3). (TIF) [file pone.0046117.s001.tif]

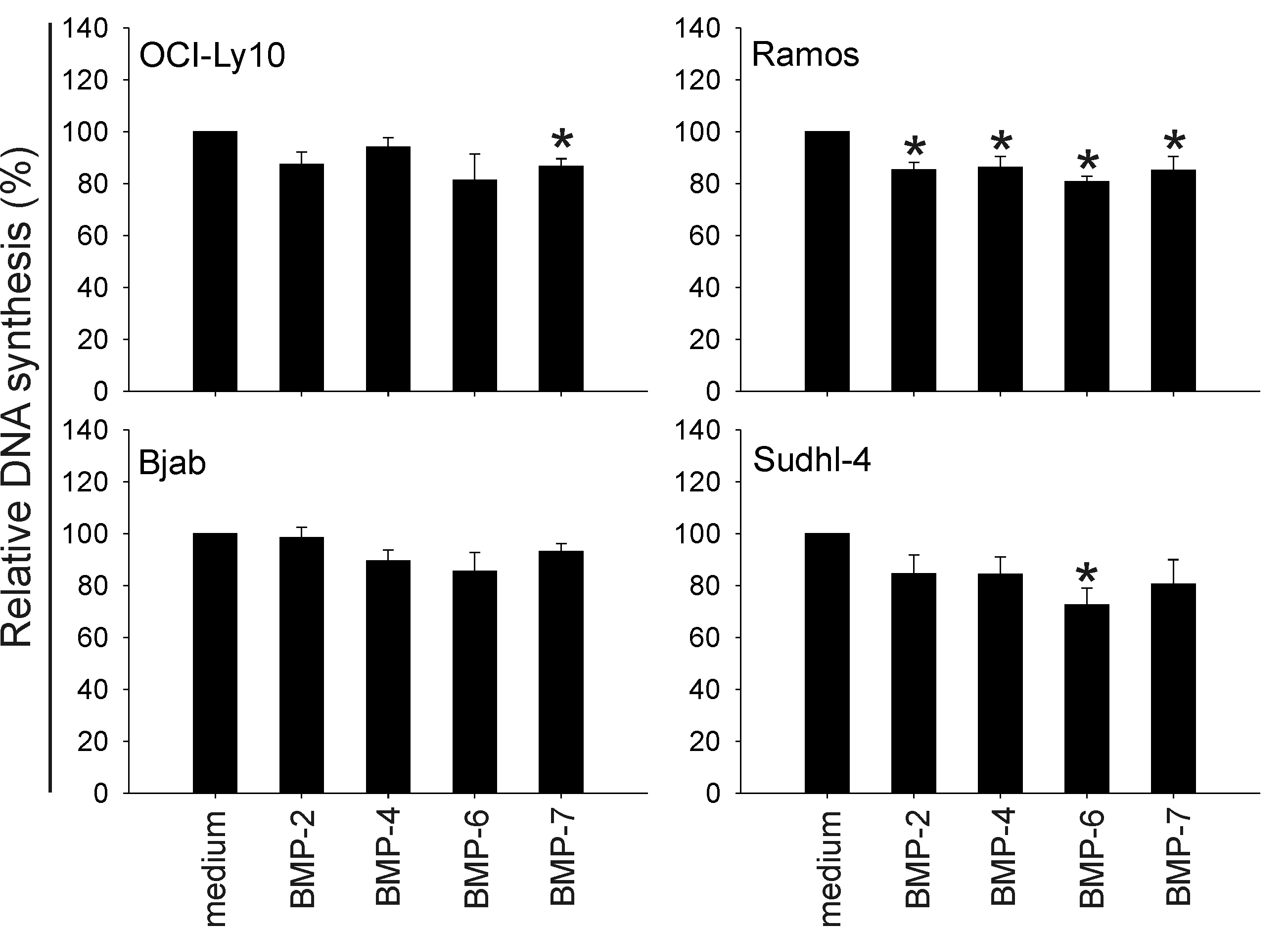

Supplement: Figure S2 — Inhibition of DNA synthesis in cell lines showing intermediate sensitivity to BMPs. Lymphoma cell lines stimulated with or without BMPs for three days before 3H-thymidine incorporation was measured. Values are obtained by normalizing mean cpm for each BMP to the mean cpm for unstimulated control in each experiment. (Means ± SEM, n = 6–7) *p<0.05. (TIF) [file pone.0046117.s002.tif]

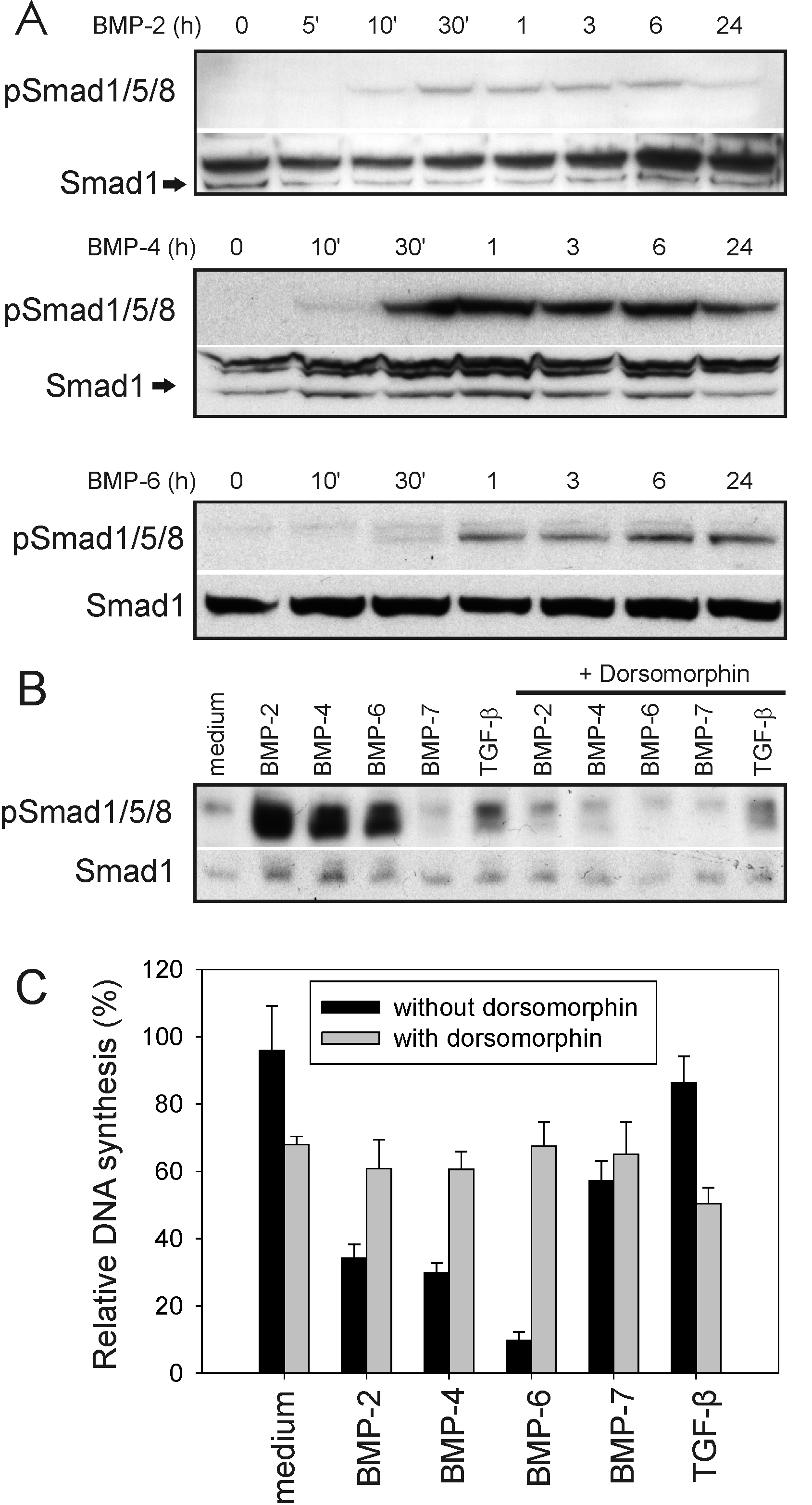

Supplement: Figure S3 — BMP-induced phosphorylation of Smad1/5/8 is selectively blocked by Dorsomorphin in sensitive Sudhl-6 cells. (A) Cells were cultured with BMP-2, -4 or -6 for different time periods, and analyzed for pSmad1/5/8 expression by Western blotting. (B) Cells stimulated with various BMPs or TGF-β with or without Dorsomorphin for one hour before cells were lysed to detect pSmad1/5/8 induction. (C) Cells were stimulated with or without various BMPs or TGF-β, in the presence or absence of Dorsomorphin for three days before 3H-thymidine incorporation was measured. (Mean cpm values ± SEM, n = 6 (n = 2 for Dorsomorphin only)). (TIF) [file pone.0046117.s003.tif]

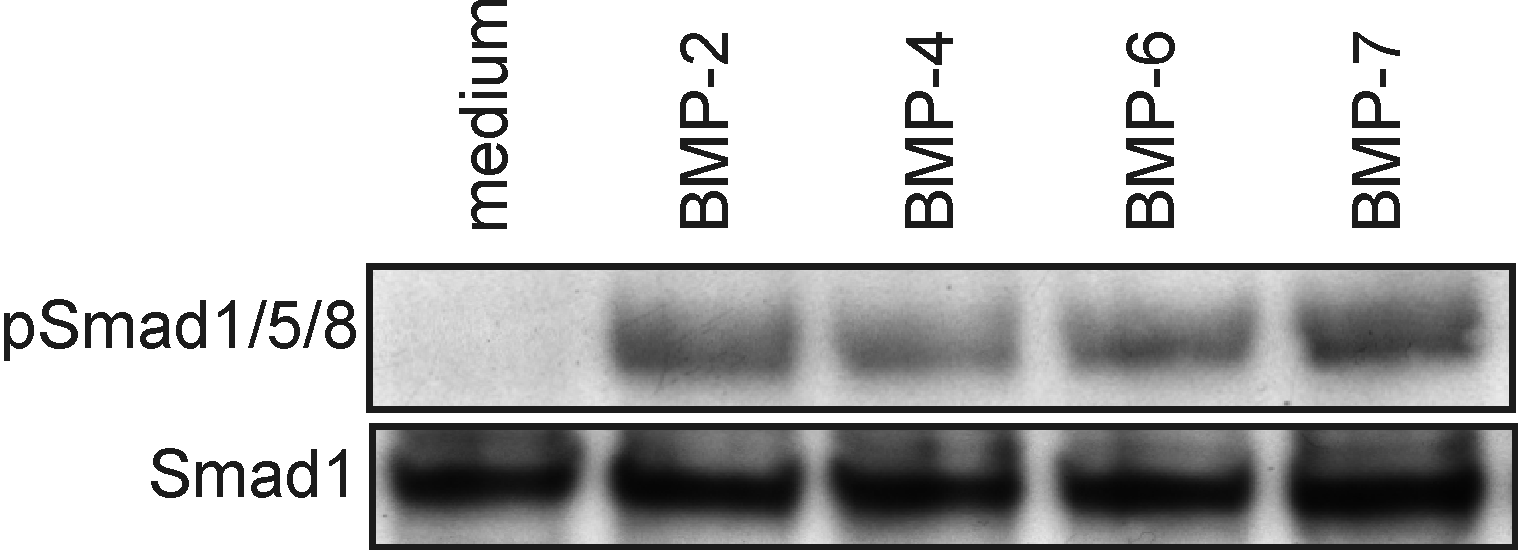

Supplement: Figure S4 — BMP-7 induces pSmad1/5/8 to the same level as other BMPs in B cells from healthy donors. CD19+ B cells were purified from peripheral blood from healthy donors and cultured with or without BMPs for one hour before pSmad1/5/8 expression was determined by Western blotting. Smad1 was used as loading control. One representative of four experiments is shown. (TIF) [file pone.0046117.s004.tif]

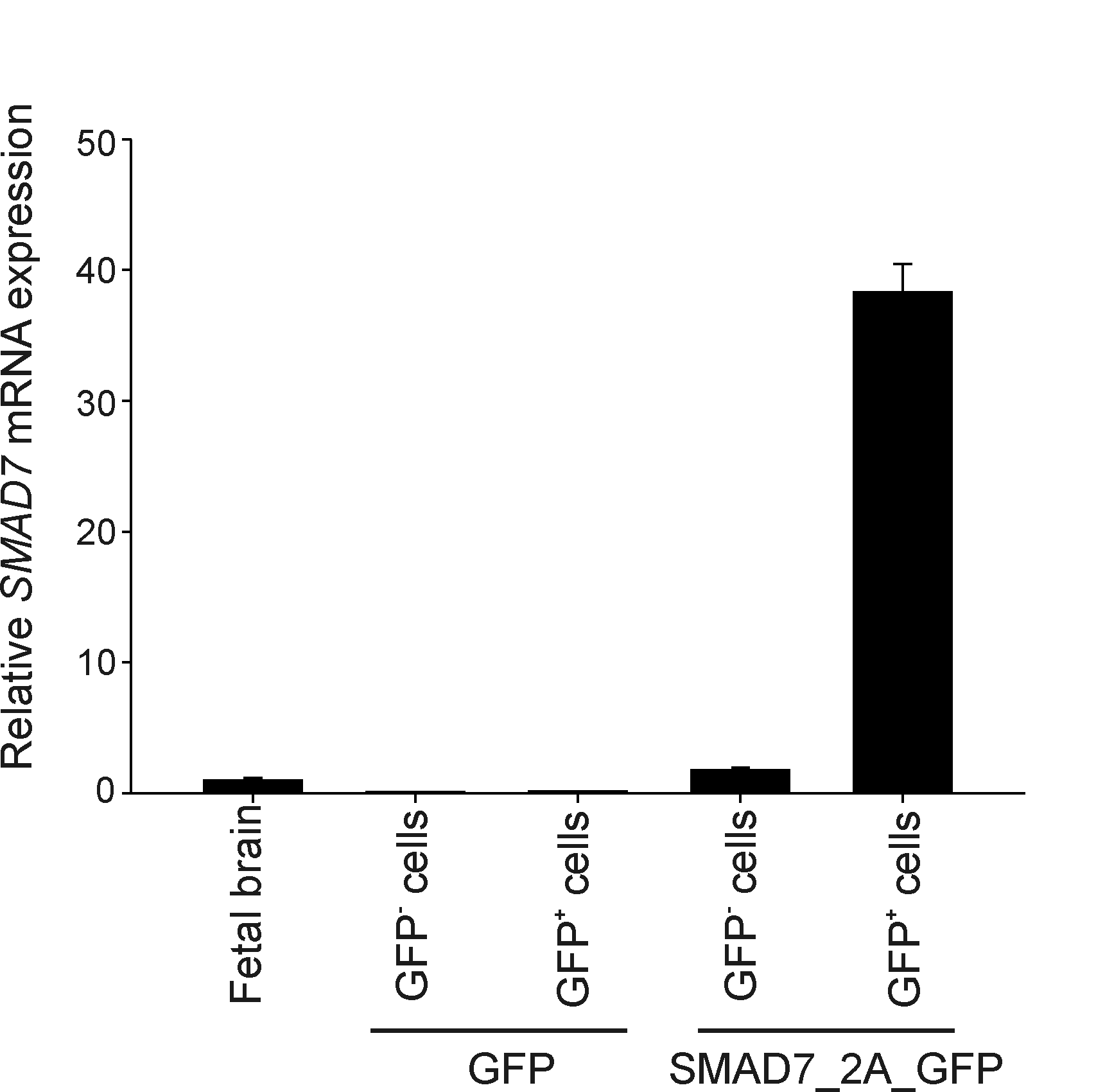

Supplement: Figure S5 — Sudhl-6 cells transduced with SMAD7_2A_GFP vector highly express SMAD7 mRNA. Real-time RT-PCR analysis of SMAD7 expression in Sudhl-6 cells which were retrovirally transduced with GFP control vector or SMAD7_2A_GFP vector and FACS sorted into GFP− or GFP+ cells. Expression is shown relative to the expression in human fetal brain tissue, and one representative of two independent experiments is shown. (TIF) [file pone.0046117.s005.tif]

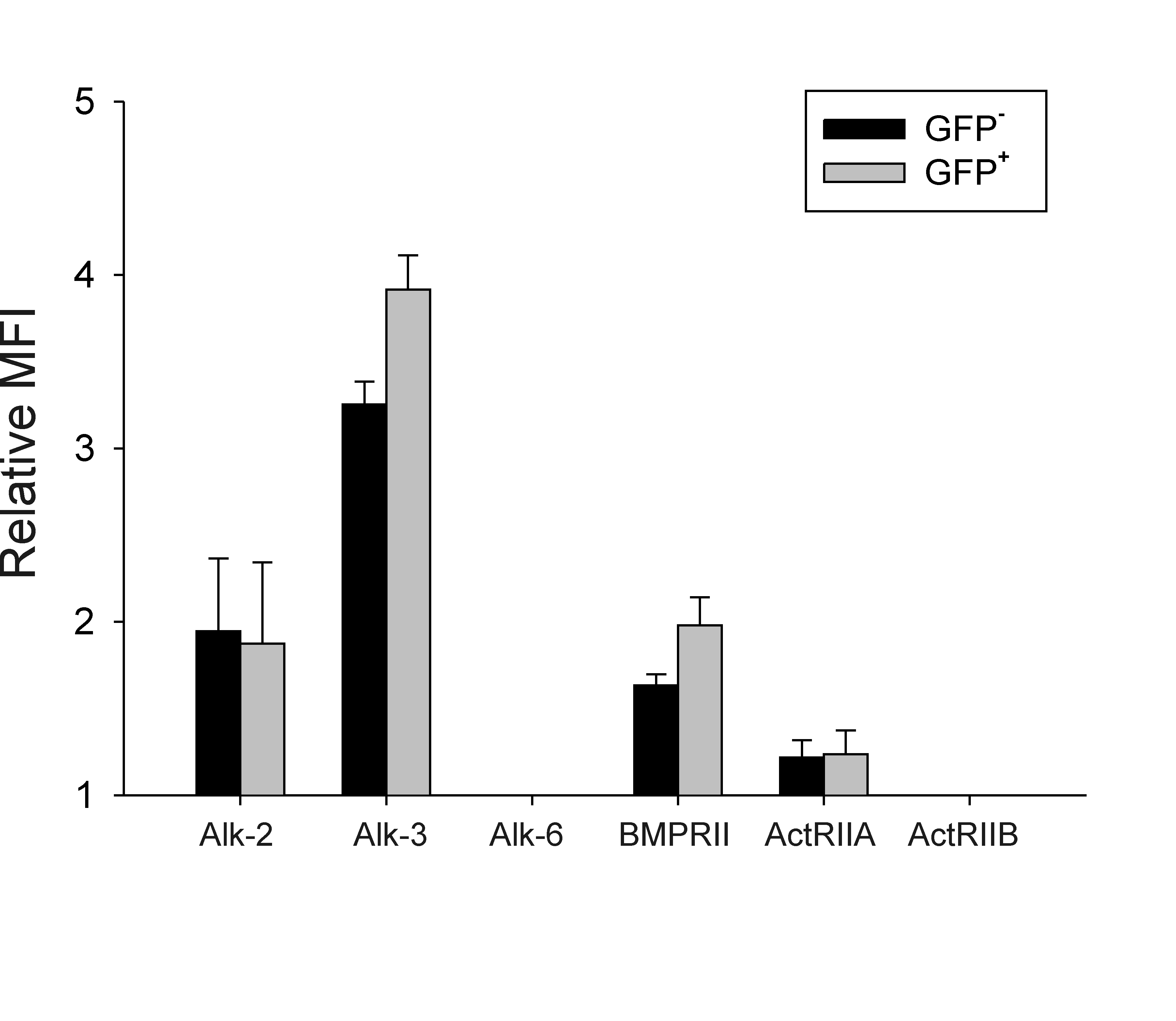

Supplement: Figure S6 — Ectopic expression of Smad7 does not alter the expression of BMP receptors. Relative BMP receptor expression in GFP− and GFP+ Sudhl-6 cells transduced with SMAD7_2A_GFP vector (means ± SD, n = 2). Values represent median fluorescent intensity (MFI) of each BMP receptor relative to the MFI of the isotype control. (TIF) [file pone.0046117.s006.tif]

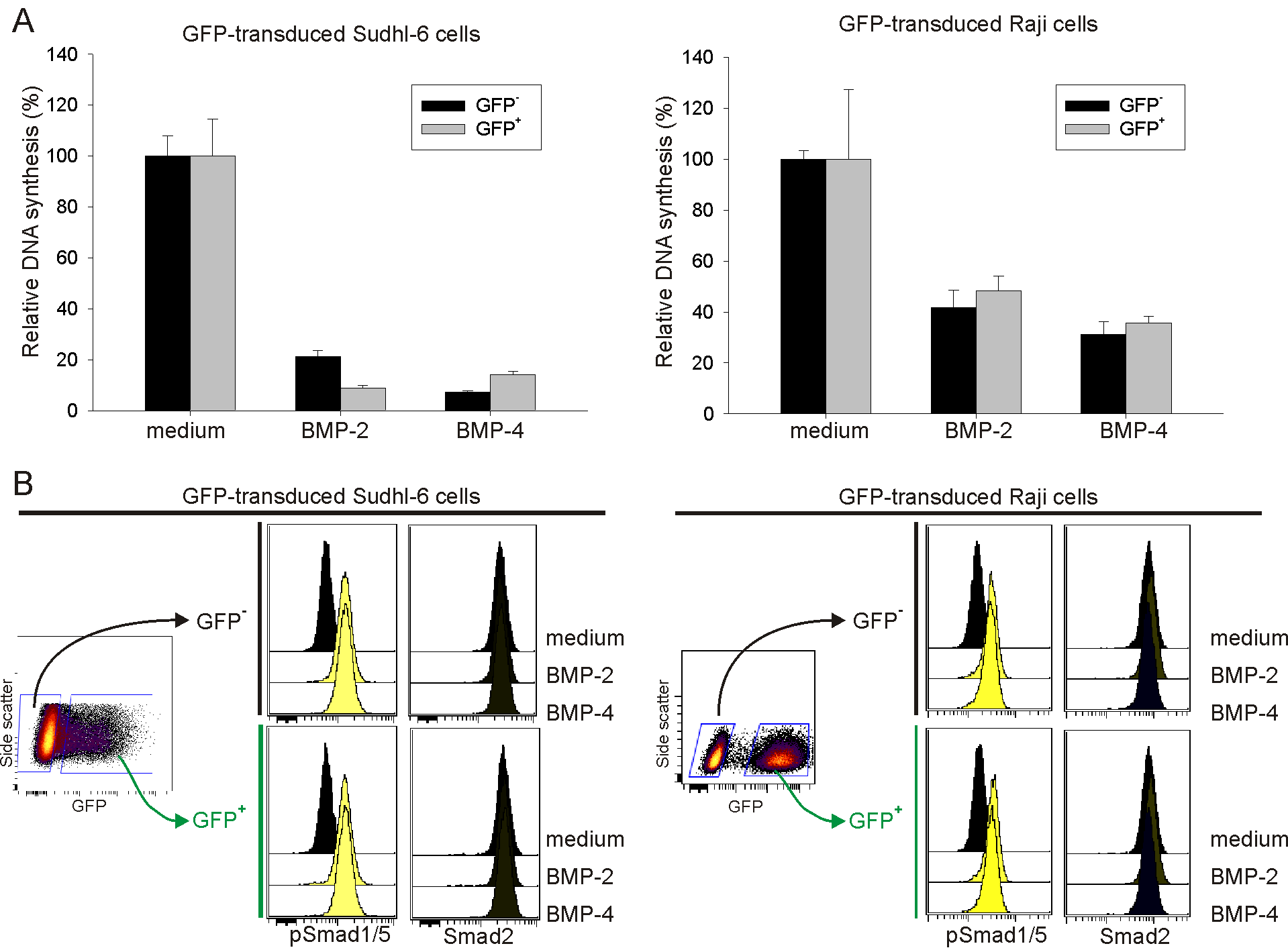

Supplement: Figure S7 — Overexpression of GFP in sensitive cell lines does not alter their sensitivity to BMPs. (A) Sudhl-6 and Raji cells were retrovirally transduced with GFP control vector, FACS sorted into GFP− or GFP+ cells, and treated with or without BMP-2 and BMP-4 for three days before 3H-thymidine incorporation was measured. Results are normalized to unstimulated control in each experiment (mean ± SD of triplicate wells). The experiments have been reproduced. (B) BMP-induced signaling was measured by treating retrovirally transduced cells with or without BMP-2 or BMP-4 for one hour, followed by detection of GFP, pSmad1/5 and Smad2 by phospho-flow cytometry. The experiments have been reproduced. (TIF) [file pone.0046117.s007.tif]

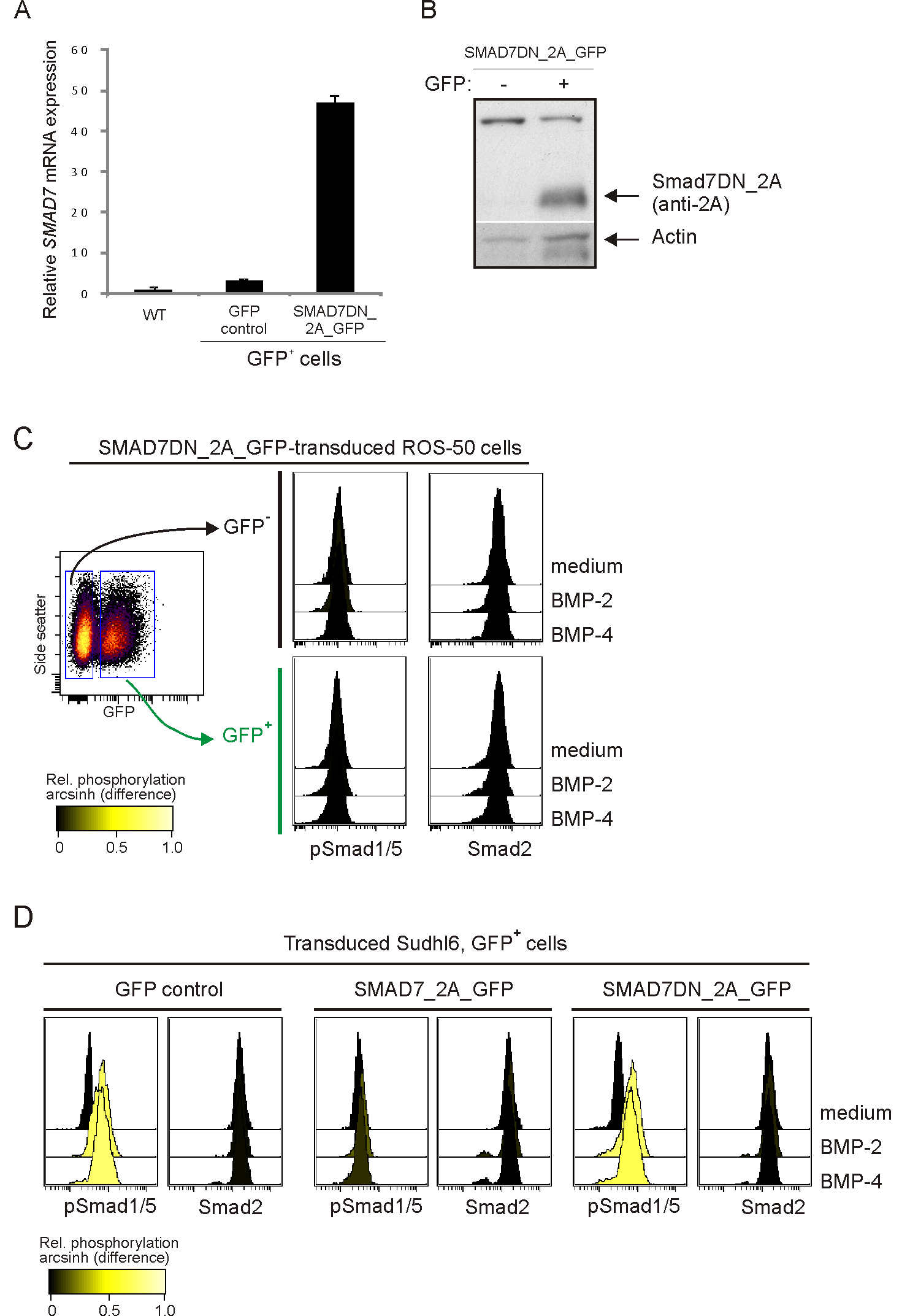

Supplement: Figure S8 — Expression of dominant negative Smad7 does not restore BMP sensitivity in ROS-50 cells. (A) ROS-50 cells transduced with SMAD7DN_2A_GFP or GFP control vector were FACS sorted, and GFP+ cells were subjected to real-time RT PCR. A gene expression assay binding to the NH2 terminus of SMAD7 (which also detects the SMAD7DN mutant) was used to measure the mRNA levels. Expression is shown relative to the expression in wild type ROS-50 cells and PGK1 is used as endogenous control. (B) Western blotting was used to measure the expression of 2A-tagged Smad7DN in GFP− and GFP+ ROS-50 cells, transduced with SMAD7DN_2A_GFP. Actin was used as loading control. (C) SMAD7DN_2A_GFP transduced ROS-50 cells were treated with or without BMP-2 or BMP-4 for one hour before they were fixed, permeabilized, stained with anti-pSmad1/5 or Smad2 and analyzed by flow cytometry (n = 2). Even though Smad7DN was highly expressed (shown in A and B), it did not alter the BMP sensitivity of ROS-50 cells. (D) To show that the Smad7DN is not a functional Smad7 protein, we transduced Sudhl-6 cells with the SMAD7DN_2A_GFP vector, in addition to the GFP control and SMAD7_2A_GFP vector (as shown in Figure 5). The cells were treated with or without BMP-2 or BMP-4 for one hour before they were fixed, permeabilized, stained with anti-pSmad1/5 or Smad2 and analyzed by flow cytometry. Results are shown for GFP+ populations only. Expression of Smad7DN did not make Sudhl-6 cells resistant as the ectopic overexpression of wild type Smad7 did. (TIF) [file pone.0046117.s008.tif]

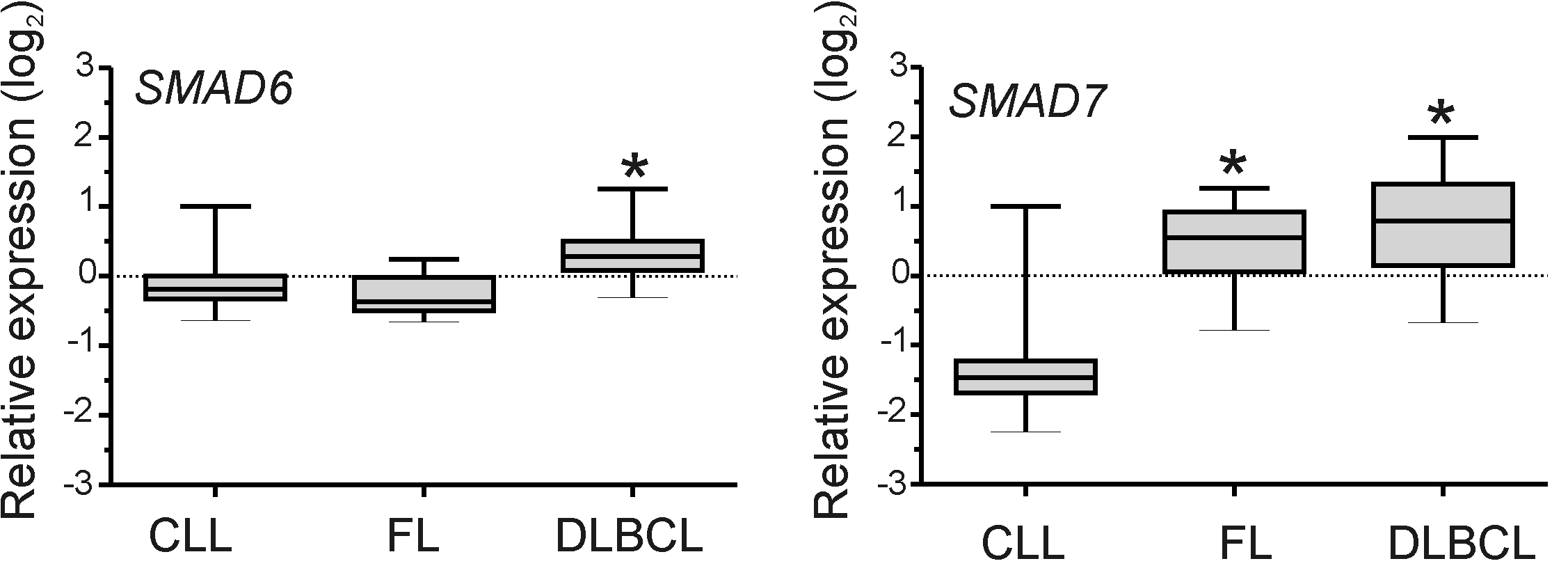

Supplement: Figure S9 — SMAD7 mRNA is higher in FL and DLBCL as compared to CLL. Relative expression of inhibitory SMADs across NHLs in the data set from Alizadeh et al. [55], * p<0.0001 compared to CLL. (TIF) [file pone.0046117.s009.tif]

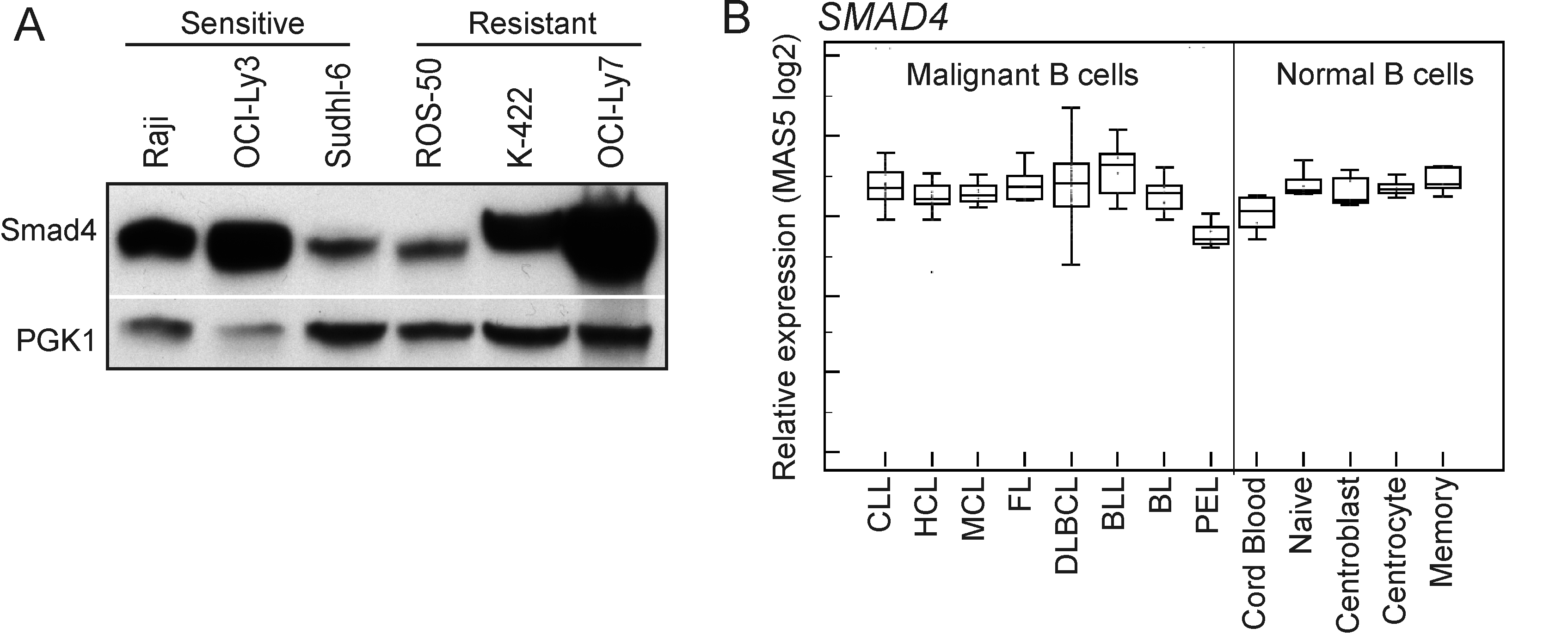

Supplement: Figure S10 — Expression of Smad4. (A) Smad4 expression in unstimulated cells, analyzed by Western blotting. Anti-PGK1 was used as loading control. Experiment was repeated once with similar results. (B) Relative expression of SMAD4 across non-Hodgkin’s lymphoma (NHL; left of line) and in normal B-cell populations (right of line) obtained from the data set of Basso et al. [16]. (TIF) [file pone.0046117.s010.tif]
