# Supplementary material for: Role of Smad Proteins in Resistance to BMP-Induced Growth Inhibition in B-Cell Lymphoma
Source: PLoS One. 2012 Oct 1;7(10):e46117. doi: 10.1371/journal.pone.0046117 (PMC3462182; doi:10.1371/journal.pone.0046117)
Supplement: Table S1 — Analysis of BMP-induced cell death in lymphoma cell lines. Lymphoma cell lines were stimulated with or without BMPs for three days before PI-positive cells were detected by FACS analysis. Means ± SEM, n = 6–7, *p<0.05. (DOC) [file pone.0046117.s011.doc]

|  |  | % PI-positive cells | | | | |
| --- | --- | --- | --- | --- | --- | --- |
|  | Cell line | medium | BMP-2 | BMP-4 | BMP-6 | BMP-7 |
| Resistant | ROS-50 | 5 ± 1 | 4 ± 1 | 4 ± 1 | 5 ± 1 | 5 ± 1 |
|  | K-422 | 6 ± 2 | 5 ± 1 | 5 ± 1 | 7 ± 2 | 5 ± 1 |
|  | OCI-Ly7 | 8 ± 2 | 9 ± 2 | 10 ± 3 | 12 ± 4 | 10 ± 4 |
| Reduced sensitivity | Bjab | 15 ± 4 | 15 ± 4 | 19 ± 4 | 17 ± 4 | 12 ± 3 |
|  | Ramos | 4 ± 0 | 5 ± 1 | 5 ± 1 | 7 ± 1 | 6 ± 1 |
|  | OCI-Ly10 | 21 ± 1 | 21 ± 1 | 20 ± 2 | 25 ± 2 | 22 ±1 |
|  | Sudhl-4 | 3 ± 1 | 4 ± 1 | 4 ± 1 | 4 ± 1 | 4 ± 1 |
| Sensitive | OCI-Ly3 | 21 ± 2 | 21 ± 2 | 20 ± 1 | 20 ± 2 | 19 ± 1 |
|  | Raji | 5 ± 1 | 6 ± 1 | 7 ± 1 | 5 ± 1 | 5 ± 1 |
|  | Sudhl-6 | 6 ± 1 | 15* ± 2 | 16* ± 1 | 31* ± 3 | 8 ± 3 |

**Table S1. Analysis of BMP-induced cell death in lymphoma cell lines.**
