# Supplementary material for: Role of Smad Proteins in Resistance to BMP-Induced Growth Inhibition in B-Cell Lymphoma
Source: PLoS One. 2012 Oct 1;7(10):e46117. doi: 10.1371/journal.pone.0046117 (PMC3462182; doi:10.1371/journal.pone.0046117)
Supplement: Table S2 — BMP-2 and -6 induce apoptosis in Sudhl-6. Sudhl-6 cells were stimulated with BMPs for three days before apoptosis was quantified with TUNEL. Means ± SEM, n = 3. (DOC) [file pone.0046117.s012.doc]

|  | % TUNEL positive cells | | |
| --- | --- | --- | --- |
|  | medium | BMP-2 | BMP-6 |
| Day 1 | 18 ± 9 | 32 ± 7 | 35 ± 4 |
| Day 2 | 11 ± 5 | 39 ± 4 | 60 ± 2 |
| Day 3 | 11 ± 3 | 39 ± 7 | 72 ± 3 |

**Table S2. BMP-2 and -6 induce apoptosis in Sudhl-6.**
