# Supplementary material for: Role of Smad Proteins in Resistance to BMP-Induced Growth Inhibition in B-Cell Lymphoma
Source: PLoS One. 2012 Oct 1;7(10):e46117. doi: 10.1371/journal.pone.0046117 (PMC3462182; doi:10.1371/journal.pone.0046117)
Supplement: Table S3 — Antibody specifications. (DOC) [file pone.0046117.s013.doc]

| Antibody | Conjugation | Dilution or final concentration | Catalog number | Manufacturer |
| --- | --- | --- | --- | --- |
| CD38 | PC5 | 8 mg/mL | A07780 | Beckman Coulter |
| IgD | PE | 1/10 | R5112 | Dako |
| Igκ | APC | 1/10 | C0222 | Dako |
| Igλ | PE | 1/20 | R0437 | Dako |
| CD3 | FITC | 1/10 | F0818 | Dako |
| CD10 | FITC | 1/10 | F0826 | Dako |
| CD3 | Pacific Blue | 1/100 | 558117 | Becton Dickinson |
| CD77 | FITC | 1/5 | 551353 | Becton Dickinson |
| CD20 | PerCPCy5.5 | 1/5 | 332781 | Becton Dickinson |
| pSmad1/5/8 | - | 1/1000 | 9511S | Cell Signalling Technology |
| Smad4 | - | 1/1000 | 9515 | Cell Signalling Technology |
| β-tubulin | - | 1/1000 | 2128 | Cell Signalling Technology |
| Smad1 | - | 1/1000 | 06-653 | Upstate (Millipore) |
| Smad1 | - | 1/1000 | 9743S | Cell Signalling Technology |
| Smad5 | - | 1/1000 | 9517 | Cell Signalling Technology |
| 2A peptide | - | 1/1000 | ABS31 | Millipore |
| Actin | - | 1/2000 | Sc-1616 | Santa Cruz Biotechnology |
| PGK1 | - | 1/500 | Ab38007 | Abcam |
| BMP-7 | - | 0.5 μg/mL | MCA2968Z | AbD Serotec |
| ActRIA | biotinylated | 10 μg/mL | BAF637 | R&D Systems |
| BMPRIA | biotinylated | 10 μg/mL | BAF820 | R&D Systems |
| BMPRIB | biotinylated | 10 μg/mL | BAF505 | R&D Systems |
| BMPRII | biotinylated | 10 μg/mL | BAF811 | R&D Systems |
| ActRIIA | biotinylated | 10 μg/mL | BAF340 | R&D Systems |
| ActRIIB | biotinylated | 10 μg/mL | BAF339 | R&D Systems |
| Antibodies used for phospho-flow cytometry | | | | |
| pErk | Alexa488 | 1/10 | 612592 | Becton Dickinson |
| pp38 | Alexa647 | 1/10 | 612595 | Becton Dickinson |
| pSmad1/5 | - | 1/200 | 9516S | Cell Signalling Technology |
| Smad2 | - | 1/400 | 86F7 | Cell Signalling Technology |
| Donkey anti-Rabbit IgG | DyLight649 | 1/200 | 711-495-152 | Jackson ImmunoResearch Laboratories, Inc |

**Table S3. Antibody specifications.**
