# Supplementary material for: Role of Smad Proteins in Resistance to BMP-Induced Growth Inhibition in B-Cell Lymphoma
Source: PLoS One. 2012 Oct 1;7(10):e46117. doi: 10.1371/journal.pone.0046117 (PMC3462182; doi:10.1371/journal.pone.0046117)
Supplement: Methods S1 — (DOC) [file pone.0046117.s014.doc]

**Supplementary methods**

**Phospho-flow cytometry**

The cells were left in medium (RPMI 1640 w/10% FCS) or treated with BMP-2, BMP-4, BMP-6 or BMP-7 as indicated for 60 min. Signaling was stopped by adding paraformaldehyde (Electron Microscopy Sciences) to a final concentration of 1.6% for 5 min, followed by wash in PBS and permeabilization in freezer-cold methanol (>90% as final concentration), and stored at -80 °C. Cells were rehydrated by washing in PBS, and stained with antibodies as specified.

**Determination of cell death and apoptosis**

Cells were cultured for 3 days and stained with 5 µg/ml propidium iodide (PI; Invitrogen) or TUNEL (Roche) according to the manufacturers’ recommendations. The cells were analyzed on a FACS Calibur (BD).

**Gene expression data**

*SMAD* mRNA expression data (log_2_ transformed) were analyzed across different lymphoma groups in the microarray dataset from Alizadeh et al.,^1^ GSE60, and included samples from patients with Chronic lymphocytic leukemia (CLL; *n* = 29), DLBCL (*n* = 42) and FL (*n* = 9).

Reference List

1. Alizadeh AA, Eisen MB, Davis RE et al. Distinct types of diffuse large B-cell lymphoma identified by gene expression profiling. Nature 2000;403:503-511.
